# Explaining changes in educational disparities in competent maternal health care services in urban and rural areas in Ethiopia

**DOI:** 10.3389/fpubh.2024.1332801

**Published:** 2024-04-12

**Authors:** Gebretsadik Shibre, Wubegzier Mekonnen, Damen Haile Mariam

**Affiliations:** ^1^Department of Reproductive, Family and Population Health, School of Public Health, Addis Ababa University, Addis Ababa, Ethiopia; ^2^Department of Health Systems Management and Health Policy, School of Public Health, Addis Ababa University, Addis Ababa, Ethiopia

**Keywords:** educational disparity, change of disparity, maternal health care services, decomposition, Ethiopia

## Abstract

**Introduction:**

Aggregate statistics of maternal health care services have improved in Ethiopia. Nevertheless, the country has one of the lowest Universal Health Coverage (UHC) service coverage indices, with slight improvement between 2000 and 2019. There are disparity studies that focus on a single dimension of inequality. However, studies that combine multiple dimensions of inequality simultaneously may have important policy implications for closing inequalities. In this study, we investigated education inequalities in the receipt of maternal health care services in rural and urban areas separately, and we examined whether these inequalities decreased, increased, or remained unchanged.

**Methods:**

The data for the study came from the 2011 and 2016 Ethiopia Demographic and Health Surveys. Using women's education as a dimension of inequality, we separately analyzed inequalities in maternal health care services in urban and rural settings. Inequalities were measured through the Erreygers concentration index, second differences, and Relative Index of Inequality (RII). Whether inequalities changed over time was analyzed by relative and absolute measures. An Oaxaca-type decomposition approach was applied to explain changes in absolute disparities over time.

**Results:**

There were glaring educational disparities in maternal health care services in urban and rural areas, where the services were more concentrated among women with better schooling. The disparities were more severe in urban than in rural areas. In urban areas, skilled birth service was the most unequal in both periods. Disparities in rural places were roughly similar for all services except that in 2011, postnatal care was the least unequal, and in 2016, skilled birth was the most unequal services. Trend analyses revealed that disparities significantly dropped in urban by absolute and relative measures. Conversely, in rural regions, the disparities grew by the concentration index measure for most services. The RII and second differences presented conflicting results regarding whether the gaps were increasing, shrinking, or remaining the same.

**Conclusion:**

Substantial disparities in maternal health care services remained and even increased, as in rural areas. Different and targeted strategies are needed for urban and rural places to close the observed educational inequalities in these areas.

## Introduction

Countries vowed to attain the Universal Health Coverage (UHC) target of the Sustainable Development Goals (SDG) by ensuring that sufficient quality health care is met for all people without being exposed to financial hardships. However, the global progress toward the health care service coverage dimension of the UHC has been uneven across regions, and the African region has been shown to have the poorest performance (1). Unless health interventions are designed to promote equity, efforts to attain UHC may improve the national average of service coverage while inequalities worsen simultaneously (2). The literature showed that monitoring inequalities are needed to address unfair disparities, identify vulnerable population subgroups left behind, and help inform equity-oriented policies that can narrow existing inequalities (3). Worldwide, evidence has accumulated on disparities in receiving competent maternal health care services by many dimensions of inequality, including wealth quintiles, location, education, maternal age, and women's empowerment (4–10).

Globally, Ethiopia has one of the lowest UHC Service Coverage Indices (SCI) even by the standard of the African region and has been making progress very slowly, with only 12 points gained in UHC SCI between 2000 and 2019 (11). Despite some improvement in aggregate maternal health care indicators at the national level and sub-nationally, evidence reveals that maternal health care inequalities remain across various social factors (12–14), highlighting the importance of solid social policies to prevent health care disparities. The government tries to improve health care equity by creating policies and strategic documents (15, 16). However, these policy changes have not been translated into massive gains in maternal health care services, and the failure to do so may be linked with suboptimal political commitment. For example, government health expenditure accounts for only 32.3% of the total health expenditure, and an unacceptably high amount of health care spending is borne from out-of-pocket expenses (17). While maternal health care services are designed for free, financial barriers have been shown to hold women back from getting skilled maternal health care in Ethiopia (18).

Since improvement in health care equity would translate into health gains, health disparities investigators continue placing an immense emphasis on comprehending how health care disparities arise in health care provisions and how to get rid of such inequalities (19). Despite disparities in maternal health care utilization globally, prior studies show that disparities are more severe in Southern East Asia and sub-Saharan Africa (20). Ethiopia is not exception to this reality, with noticeable socioeconomic and geographical gradients in the utilization of skilled maternal health care services (19).

Even though studies addressing health care disparities involving social factors exist, studies doing finely grained analyses of maternal health care equity in Ethiopia are limited. For example, it is not enough to investigate differences in maternal health care between urban and rural areas as they are heterogeneous populations. Instead, equity analyses should be done in urban and rural settings separately by relevant equity stratifiers to understand the unique experiences of various subpopulations within urban and rural settings. World Health Organization (WHO) highlights that sometimes it would be necessary to simultaneously combine multiple dimensions of inequality, such as women's education and residence, to analyze disparities more deeply. This would help identify a situation where educational inequality may be high in urban settings but low in rural areas, and this finding may have important policy implications, such as monitoring educational disparities in urban and rural areas separately (21). The study aims to analyze educational disparities in urban and rural settings and explain how the disparities have changed over time due to changes in the social determinants of health.

## Materials and methods

### Data source and sample

The data for this study came from the Ethiopia Demographic and Health Surveys (EDHS) conducted in 2011 and 2016. They are nationally representative household surveys intended to gather information on demographics, health, and nutrition pertaining to various topics, such as the provision of maternity health care (22). The surveys chose their samples using a stratified two-stage cluster sampling approach. Samples were chosen in two stages independently from each stratum after dividing the country into strata created by the cross-classification of regions and urban-rural residence. First, a frame from the 2007 Ethiopia Population and Housing Census was used to select 624 and 645 primary sample units (PSUs) in 2011 and 2016, respectively, using probability proportional to the PSU size. In the second step, 30 and 28 households were systematically chosen from each PSU for the 2011 and 2016 EDHS surveys respectively. Women in the reproductive years who slept the night before the surveys in the chosen households were qualified to participate. For the 2011 and 2016 surveys, 16515 and 15683 women were interviewed, with 95.0% and 94.6% response rates, respectively. Missing and do not know responses were handled based on the 2018 guide to DHS statistics (22). A detailed description of the methodology of the surveys can be found elsewhere (23, 24).

### Outcome measures

In this study, five maternal health services were assessed and included ANC quality, early ANC, receipt of ANC at least four times (ANC4+), skilled birth (SBA), and postnatal care for mothers within 2 days of birth (PNC). The measures represent five binary variables, each taking values of 1 and 0, with 1 indicating receipt of services and 0 otherwise. ANC quality was defined based on whether three services, namely, blood pressure measuring and urine and blood testing, were provided to women who had at least one ANC visit with a competent health care worker. If a woman obtains all these three services, ANC quality is coded 1 and 0 otherwise. Early ANC was coded 1 if a woman had her first ANC within the first trimester and 0 if not. ANC4+ was coded 1 if a woman had ANC visits at least four times and 0 if she had three or fewer visits. SBA was dichotomized as having birth attended by skilled health professionals, coded 1, and birth not attended by qualified providers, coded 0. Finally, PNC was coded 1 if a woman received care within 2 days of delivery and 0 otherwise. All the measures of maternal health care represent services provided by skilled health professionals, which include doctors, nurses, midwives, and health officers. According to the World Health Organization, a skilled birth attendant is a health care worker who receives the education needed to attend a pregnancy, delivery, and postpartum period and recognize, manage, and refer complications (25).

### Dimension of equity

Dimensions of equity, also known as equity stratifier, is a variable used to measure disparities in health and health care where that disparity may be considered unfair (26). In this study, maternal education represents a dimension of equity with which disparities in maternal health care services were examined. In the literature, women's education has been shown to improve the utilization of maternal health care services (27, 28) and improve health and wellbeing. It also relates to all SDGs, including SDG 3 (29). Three categories—no education, primary school, and secondary/higher—were used to categorize women's education.

### Factors

Based on theoretical and empirical significance in the literature (30, 31), individual, household level, and community factors have been chosen in the study to be used to explain educational disparities in maternal health care services. Age (at pregnancy for ANC measures and at birth for SBA and PNC), women's education, partner education, religion, media exposure, women and partner occupations, women's empowerment, and birth order were individual-level factors. The age of women was dichotomized into adolescent (< 20 years) and non-adolescent (>=20 years) groups. The educational status of a woman's spouse was categorized as no education, primary, secondary, and higher education. The religions of the women included Orthodox, Protestant Christian, Muslim, and others. Media exposure was measured using the information on exposure to newspaper reading, watching TV, and listening to the radio. The variable was measured as binary, where 1 indicates that a woman was exposed to at least one of the media and 0 otherwise. Spousal occupations were counted as having an occupation and not having an occupation in the 12 months preceding the respective surveys. Women's empowerment was measured using a validated index, SWPER Global, following a previous study. The index was intended to measure women's empowerment in the context of LMICs. The index has three domains that measure three areas of empowerment: attitude toward violence, social independence, and decision-making. An extended discussion on the index, including the Stata codes used to compute it, can be found elsewhere (32). In addition, the Appendix includes a brief overview of the index. Household socioeconomic status was proxied by wealth quintile (33) and was recorded as binary (richest vs. non-richest) in urban due to sample problems. It was used as it is in rural areas, where it has five quintiles. The region is the only community-level variable used in the study. It included nine provinces and two city administrations.

### Statistical analyses

Three measures of inequality, namely, ratio of Relative Indices of Inequality (rRII), second differences of average marginal effects, and Concentration index (C), were used to quantify the extent of educational disparities in receipt of maternal health care services. The choice of the inequality measures in the study is in line with suggestions that relative and absolute inequality measures be applied in an investigation because they complement each other (21) and that different measures assess different aspects of inequality (34).

The Relative Index of Inequality is a regression-based, relative, and complex measure of inequality (34). The Relative Index of Inequality is used to depict the gradient of health care indicators across ranked categorical variables such as socioeconomic status. It is the ratio in predicted values of a health indicator between women with, say, the highest level of education and those with the lowest level of education while accounting for the whole distribution of education using a suitable regression model.

The Relative Index of Inequality was computed using a modified Poisson regression model through the generalized linear regression with the “Poisson” family and “log” link, where the overestimation of variances was rectified by estimating robust error (35). The ranking variable, women's education, was first converted into one suitable for calculating the Relative Index of Inequality using the ridit analyses, where each category of education was assigned a ridit score. Proposed by Bross in 1958, ridit is a statistical method used to quantitatively analyze ranked categorical data (36). We used the Stata command *wridit* to compute the ridit score (37). A discussion of how the Relative Index of Inequality is calculated can be found elsewhere (38).

The over time change in disparity was estimated by doing an interaction between the ridit-converted women's education and the year of the surveys, where the exponentiated coefficient of the interaction term indicates the ratio of Relative Indices of Inequality, hereafter called rRII, measuring the disparity between the two time periods. The binary variable “year” in the interaction analysis was assigned a value of 2 for the year 2016 and a value of 1 for the year 2011. When the multiplicative interaction effect, as measured by the rRII, is < 1, it suggests a reduction in disparity in the second period compared to that of the first period. If it exceeds 1, the disparity widens over time. Estimation of the rRII for each outcome was adjusted for the effect of several important factors mentioned above under the “factors” subsection.

Second differences of average marginal effects were estimated using *Stata's mtable* and *mlincom* commands, following a logit model that included interaction between women's education and survey period alongside all other factors. Literature suggests using a second difference in average marginal effect for interpreting interaction terms in a non-linear model (39). The Appendix offers more details on the second differences and the Stata line of codes demonstrating their computation. We must adhere to conventional procedures, including the use of multiplicative and marginal interaction effects, to prevent potential misinterpretation of the results (40).

The standard concentration index (C) is a complex and relative measure of disparity in a variable over the distribution of another (Kakwani, 1977, as cited in O'Donnell O et al., 2016) (41). C is used to measure socioeconomic-related health inequality (Wagstaff et al., 1991; O'Donnell et al., 2008, as cited in O'Donnell O et al., 2016) (41), which is expressed as follows:


(1) $$\begin{array}{l} C=\frac{2}{n\mu h}\sum_{i=1}^{n}\left(hiRi-1\right) \end{array}$$

Where n is the sample size, h*i* is the value of the individual i's health variable (e.g., a measure of maternal health service), μh is the mean of the measure, and R*i* is the individual i's fractional rank in the distribution of a ranking variable (e.g., socioeconomic status). Values for C range from −1 to 1, inclusive. A negative value denotes a disproportionately higher concentration of an outcome (such as maternal health care services) among disadvantaged positions (e.g., lack of education), while a positive value indicates that the better-off (e.g., higher levels of education) subgroups account for a large portion of the outcome.

Research indicated that outcomes bounded and not measured on a ratio scale, such as the maternal health care service, should not have the standard C calculated. The rationale is that when the C is computed for the bounded indicators, the values do not lie within the range of−1 and 1. Various variants of C have been proposed in the literature for bounded outcomes, and for this investigation, the Erreygers normalized concentration index (ECI) was utilized to overcome the C's deficiency (42). The ECI measures absolute inequality and has desirable properties, according to Erreygers, including mirror property, which means that the ECI of health and ill-health are mirror images of each other (Erreygers, 2009a, as cited in Kjellsson G et al., 2013) (42). Using the C in Equation (1), the ECI can be written in Equation (2) as follows:


(2) $$\begin{array}{l} ECI=4\mu h\times C \end{array}$$

Where μh refers to the mean of each of the outcomes in the study. The ECI was estimated using the Stata command, *conindex* (41).

### Decomposition analyses

The ECIs were computed for the 2011 and 2016 surveys separately and were decomposed to examine the contribution of various factors to the educational disparities in maternal health care services. The regression method that O'Donnell O et al. suggested was applied to do the decomposition analyses (43). A generalized linear model was fitted with a binomial family and logit link function. The regression analyses present the elasticity, concentration index, and percentage contribution of each of the factors entered into the models. Elasticity measures the degree of responsiveness of maternal health care service utilization to changes in the values of the factors. For example, what would happen to the ANC utilization when the place of residence of women changes from urban to rural or vice versa? The concentration index of the factors indicates how unequally the factors are distributed across the categories of women's education. Percentage contribution shows the amount of contribution each factor has on the observed educational inequality in the use of the services.

Finally, an Oaxaca-type decomposition approach was used to explain changes in educational inequalities over time (43). This decomposition approach allows us to decompose the changes in education-related inequalities in health care services into changes in inequality in the factors and changes in the elasticities of the services concerning these factors. Further details on the Oaxaca-type decomposition are given in the Appendix. The Oaxaca-type decomposition was computed using the following equation:


(3) $$\begin{array}{l} \Delta ECI=\sum_{K}\eta \text{kt}(\text{Ckt}-\text{Ckt}-1)+\sum_{K}\text{Ckt}-1(\eta \text{kt}\\ \;-\eta \text{kt}-1)+\Delta \;(\text{GC}\varepsilon \text{t}/\mu ) \end{array}$$

Where η*k* and Ck indicate elasticities and concentration indices of k factors, t indicates the 2016 survey, t – 1 indicates the 2011 survey, and Δ denotes the first differences. GC is a generalized concertation index for error terms. Equation (3) presents the summation of the difference in C (*Ckt*−*Ckt*−1) weighted by the elasticities of the determinants in 2016 and the difference in elasticities (η*kt*−η*kt*−1) weighted by the concentration indices of the determinants in the first survey, 2011. All analyses accounted for the survey characteristics, namely clustering, stratification, and unequal selection probabilities. All analyses were carried out in Stata v16.

## Results

### Distribution of maternal health care services by women's education

In both periods, the Erreygers Concentration Index (ECI) is positive for all five health care services in both urban and rural locations, indicating varying levels of disparity across these services. As an example, in the rural setting in 2011, the health care service with the highest disparity based on the ECI was SBA, with a value of 0.44 (95% confidence interval: 0.33, 0.55). This was followed by ANC4+ with a value of 0.35 (95% confidence interval: 0.28, 0.42), while early ANC had the lowest ECI with a value of 0.16 (95% confidence interval: 0.08, 0.24). The results reveal that there is a greater concentration of services among women who achieved higher levels of education, as shown in Table 1 and Supplementary Figure.

**Table 1:** Erreygers concentration indices, second differences, and relative indices of inequality for the maternal health care services, 2011–2016 EDHS.

**Variables**	**Urban**				**Rural**			
	**ECI (95% CI)**		**rRII (95%CI)**	**Second difference (95% CI)**	**ECI (95% CI)**		**rRII (95%CI)**	**Second difference (95% CI)**
	**2011**	**2016**	**2011–2016**		**2011**	**2016**	**2011–2016**	
ANC quality	0.23 (0.14, 0.32)	0.12 (0.03, 0.21)	0.59 (0.39, 0.89)	−0.091 (−0.262, 0.079)	0.10 (0.04, 0.16)	0.11 (0.05, 0.17)	0.62 (0.36, 1.07)	−0.034 (−0.258, 0.190)
Early ANC	0.16 (0.08, 0.24)	0.12 (0.02, 0.22)	0.57 (0.29, 1.14)	−0.088 (−0.232, 0.056)	0.04 (0.02, 0.06)	0.08 (0.05, 0.11)	0.59 (0.23,1.52)	−0.036 (−0.169, 0.097)
ANC4+	0.35 (0.28, 0.42)	0.18 (0.07,0.29)	0.38 (0.24, 0.60)	−0.227 (−0.366, −0.087)	0.08 (0.05, 0.11)	0.12 (0.09, 0.15)	0.55 (0.29, 1.07)	−0.015 (−0.170, 0.141)
SBA	0.44 (0.33,0.55)	0.32 (0.22,0.42)	0.37 (0.20,0.69)	−0.098 (−0.257, 0.061)	0.04 (0.02,0.06)	0.17 (0.14,0.20)	0.58 (0.24,1.36)	0.062 (−0.049, 0.173)
PNC	0.23 (0.12, 0.34)	0.08 (−0.03, 0.19)	0.23 (0.09, 0.56)	−0.289 (−0.517, −0.061)	0.02 (0.004, 0.04)	0.1 (0.06, 0.14)	0.3 (0.05, 1.77)	−0.073 (−0.234, 0.089)

ECI: Erreygers Concentration Index; 95%CI: 95% confidence interval; AME: Average Marginal Effect.

### Change in the educational disparities in maternal health care services

As time passed, urban and rural areas' educational gaps in health care developed differently. The rRII revealed that except for the disparity in early ANC, where there is a mix of decreasing, unchanged, and increasing patterns, inequalities in obtaining the services in urban regions considerably dropped. For instance, the rRII demonstrated the most substantial decline in disparity for PNC with an rRII of 0.23 (95% confidence interval: 0.09, 0.56). This equates to a reduction between 44% and 91%, with a considerable 77% reduction based on the point estimate. In contrast, ANC quality unveiled the lowest decrease with an rRII of 0.59 (95% confidence interval: 0.39, 0.89). This indicates a drop in inequality ranging from 11% to 61%, with the point estimate indicating a 41% decrease. Similarly, the ECIs suggested a reduction in disparities over time, except for early ANC, where the disparity remained stable based on the 95% confidence interval despite a decrease in its point estimate. For example, the ECI of the ANC4+ fell from 0.35 (95% confidence interval: 0.28, 0.42) in 2011 to 0.18 (95% confidence interval: 0.07, 0.29). Finally, the second difference demonstrated a decrease in inequalities in ANC4+ with the second difference of −0.227 (95% confidence interval: −0.366, −0.087) and PNC with the second difference of −0.289 (95% confidence interval: −0.517, −0.061). However, the disparities in the other health care services fell based on point estimates, and their confidence intervals indicated different patterns of decrease, increase, and stability. The SBA, for example, had the second difference of −0.098 (95% confidence interval: −0.257, 0.061), suggesting a decrease, increase, and stable patterns. See Table 1 for the details.

In rural places, the inequalities measured by the ECIs, by and large, increased, except for the ANC quality, which remained unchanged. Particularly, PNC and ANC4+ services showed the largest worsening of inequalities over time. Specifically, in 2011, the ECI for PNC was 0.02 (95% confidence interval: 0.004, 0.04), which rose to 0.1 (95% confidence interval: 0.06, 0.14) in 2016. Similarly, the ECI for ANC4+ was 0.04 (95% confidence interval: 0.02, 0.06) in 2011 and increased to 0.17 (95% confidencev interval: 0.14, 0.20) in 2016. However, there were contradicting findings from the rRII and a second difference on whether the disparities in maternal health care services were widening, contracting, or remaining the same. The rRII showed, for instance, that the PNC had an rRII of 0.3 (0.05, 1.77). This indicates that although the point estimate shows a 70% reduction in the disparity, the interval indicates that the change involved an increase, decline, and stabilization of the disparity. See Table 1 for the details.

Supplementary Tables 1–5 are decomposition findings for each year separately. Although we are interested in the disparity change over time, the survey-specific findings facilitate interpreting results of the decomposition of change. We found that the contributions of variables to the disparity increased, decreased, or remained unchanged between the two surveys, and the dynamics of this changing influence of the variables are related to the change in their elasticities and concentration indices. Education (both the mother and partner), women's empowerment, and birth order are among the decisive drivers of inequalities in both years. From the regions, Addis Ababa and Amhara regions had more remarkable contributions to most of the disparities, and their influences decreased over time. See Supplementary Tables 1–5 for detailed information on the survey-specific analyses of inequalities.

How did the changes in the factors contribute to the decline (in urban areas) and rise (in rural areas) in the concentration index-related inequalities in health care services? Findings presented in Tables 2–6 from the Oaxaca-like decomposition of change showed that changes in the disparities were associated with changes in the elasticities (impacts) and concentration indices (degree of spread or distribution) of the factors considered in the study. All of the variables included in Table 2 through 6 contributed to the change in the disparities, albeit to differing degrees and signs. The contribution of each variable to the disparities' narrowing or widening depended on whether the variables' elasticities and concentration indices rose or decreased over time. Accurate interpretation of the factors contributing to the change in disparities in maternal health care services requires a careful understanding of the concentration indices of the health care services (Table 1), the evolution of the concentration indices and elasticities of the exposure variables (Tables 2–6), and finally, the concentration indices and elasticities of the exposure variables for each survey (Supplementary Tables 1–5).

**Table 2:** Factors contributing to the change in educational disparities in ANC quality, 2011–2016 EDHS.

**Variables**	**Urban**				**Rural**			
	**Change in CI**	**Change in elasticity**	**Total**	**%**	**Change in CI**	**Change in elasticity**	**Total**	**%**
**Age at pregnancy**								
Not adolescent (ref)								
Adolescent	−0.006	−0.005	−0.01	9.09	−0.007	0.048	0.04	400.00
**Region**								
Tigray (ref)								
Afar	0.001	−0.002	−0.002	1.82	−0.001	0.008	0.007	70.00
Amhara	−0.012	−0.029	−0.041	37.27	−0.018	−0.061	−0.078	−780.00
Oromia	−0.003	0.005	0.002	−1.82	0.056	−0.049	0.006	60.00
Somali	0	−0.003	−0.002	1.82	−0.002	0.021	0.019	190.00
Benisahangul Gumuz	0	−0.003	−0.003	2.73	−0.002	0	−0.001	−10.00
SNNP	0.026	−0.004	0.022	−20.00	−0.015	0	−0.015	−150.00
Gambela	0	0	0	0.00	0	−0.002	−0.002	−20.00
Harar	−0.001	−0.002	−0.003	2.73	0	0.001	0.001	10.00
Addis Ababa	−0.005	−0.072	−0.077	70.00		
Dire Dawa	−0.001	0	−0.002	1.82	0	0.001	0.001	10.00
**Partner's education**								
No education (ref)								
Primary	−0.06	0.012	−0.048	43.64	−0.003	0.082	0.08	800.00
Secondary	−0.011	−0.024	−0.035	31.82	0	0.035	0.036	360.00
Higher	−0.079	0.116	0.037	−33.64	−0.002	−0.022	−0.024	−240.00
**Religion**								
Orthodox (ref)								
Protestant	0.017	−0.01	0.007	−6.36	−0.005	0.034	0.029	290.00
Muslim	0.001	−0.009	−0.008	7.27	−0.002	−0.023	−0.025	−250.00
Other	−0.008	−0.002	−0.011	10.00	−0.001	−0.005	−0.006	−60.00
**Media exposure**								
Not exposed (ref)								
Exposed	0.002	−0.037	−0.036	32.73	0.002	−0.044	−0.043	−430.00
**Women's education**								
No education (ref)								
Primary	0.02	0.086	0.107	−97.27	−0.004	−0.327	−0.332	−3320.00
Secondary or higher	0.052	−0.463	−0.411	373.64	−0.005	−0.143	−0.148	−1480.00
**Women's occupation**								
No occupation (ref)								
Has occupation	−0.001	0.002	0.001	−0.91	0	0.002	0.002	20.00
**Partner's occupation**								
No occupation (ref)								
Has occupation	0	−0.003	−0.003	2.73	−0.001	−0.005	−0.006	−60.00
**Attitude toward violence**								
Low (ref)								
Medium	0.004	0.007	0.01	−9.09	0.001	−0.005	−0.004	−40.00
High	−0.013	−0.047	−0.06	54.55	0.002	−0.032	−0.03	−300.00
**Social independence**								
Low (ref)								
Medium	−0.004	−0.005	−0.009	8.18	0.002	0.049	0.051	510.00
High	−0.038	0.226	0.188	−170.91	0	0.105	0.105	1050.00
**Decision-making**								
Low (ref)								
Medium	0.004	0.002	0.006	−5.45	−0.001	0	−0.001	−10.00
High	−0.043	0.052	0.01	−9.09	0	−0.005	−0.005	−50.00
**Birth order**								
**1 (ref)**								
2-3	0	0.018	0.017	−15.45	−0.001	0.018	0.017	170.00
4-5	0.002	0.024	0.026	−23.64	0.01	0.007	0.017	170.00
6+	0.008	0.055	0.063	−57.27	0.009	−0.03	−0.021	−210.00
**Wealth (2 categories)**								
Not richest (ref)								
Richest	−0.002	0.049	0.048	−43.64				
**Wealth quintile**								
Poorest (ref)								
Poorer					0	0	−0.001	−10.00
Middle					0.004	0.008	0.012	120.00
Richer					−0.001	−0.051	−0.052	−520.00
Richest					−0.013	0.008	−0.005	−50.00
**ECI2016-ECI2011**			**−0.11**				**0.01**	

ECI2016-ECI2011 refers to the difference between the ECI of 2016 and 2011. ECI refers to the Erreygers concentration index.

**Table 3:** Factors contributing to the change in educational disparities in early ANC, 2011–2016 EDHS.

	**Change in CI**	**Change in elasticity**	**Total change**	**%**	**Change in CI**	**Change in elasticity**	**Total change**	**%**
**Age at pregnancy**								
Not adolescent (ref)								
Adolescent	−0.003	−0.007	−0.011	27.50	−0.029	0.23	0.201	502.50
**Region**								
Tigray (ref)								
Afar	−0.002	0.001	−0.001	2.50	−0.001	0.045	0.044	110.00
Amhara	0.003	0.001	0.004	−10.00	−0.035	−0.065	−0.1	−250.00
Oromia	−0.005	−0.019	−0.024	60.00	0.276	−0.123	0.153	382.50
Somali	0	0.02	0.02	−50.00	−0.01	0.083	0.072	180.00
Benisahangul Gumuz	−0.002	0.001	0	0.00	−0.009	0.002	−0.008	−20.00
SNNP	0.012	−0.045	−0.034	85.00	−0.067	−0.091	−0.158	−395.00
Gambela	0	0	0	0.00	0	−0.019	−0.019	−47.50
Harar	−0.002	−0.004	−0.007	17.50	0	0.001	0.001	2.50
Addis Ababa	−0.011	−0.051	−0.062	155.00	
Dire Dawa	−0.008	0	−0.008	20.00	0.001	0	0.002	5.00
**Partner's education**								
No education (ref)								
Primary	−0.015	0.088	0.073	−182.50	−0.006	−0.05	−0.056	−140.00
Secondary	−0.007	−0.083	−0.09	225.00	0	−0.182	−0.183	−457.50
Higher	−0.055	−0.221	−0.276	690.00	−0.001	0.204	0.203	507.50
**Religion**								
Orthodox (ref)								
Protestant	0.074	0.008	0.082	−205.00	−0.01	0.23	0.22	550.00
Muslim	0.01	−0.011	−0.001	2.50	−0.01	−0.178	−0.188	−470.00
Other	−0.007	−0.001	−0.008	20.00	0.005	−0.013	−0.008	−20.00
**Media exposure**								
Not exposed (ref)								
Exposed	0.003	−0.102	−0.099	247.50	0.013	−0.162	−0.15	−375.00
**Women's education**								
No education (ref)								
Primary	−0.041	−0.012	−0.052	130.00	−0.103	−0.323	−0.426	−1065.00
Secondary/higher	−0.003	0.433	0.43	−1075.00	−0.017	−0.538	−0.555	−1387.50
**Women's occupation**								
Has no occupation (ref)								
Has occupation	−0.001	0.003	0.002	−5.00	−0.005	−0.033	−0.037	−92.50
**Partner's occupation**								
Has no occupation (ref)								
Has occupation	0	0.033	0.033	−82.50	−0.008	−0.006	−0.014	−35.00
**Attitude toward violence**								
Low (ref)								
Medium	0.022	0.032	0.054	−135.00	0	−0.016	−0.016	−40.00
High	−0.007	−0.241	−0.248	620.00	−0.004	−0.184	−0.188	−470.00
**Social independence**								
Low (ref)								
Medium	0.009	0.016	0.025	−62.50	−0.008	−0.002	−0.01	−25.00
High	−0.031	−0.25	−0.281	702.50	0	0.219	0.219	547.50
**Decision-making**								
Low (ref)								
Medium	0.014	0.083	0.097	−242.50	−0.009	0.002	−0.007	−17.50
High	−0.053	−0.07	−0.123	307.50	−0.008	0.04	0.033	82.50
**Birth order**								
1 (ref)								
2-3	0.005	−0.07	−0.064	160.00	−0.006	0.16	0.154	385.00
4-5	0.003	0.074	0.077	−192.50	0.04	−0.034	0.006	15.00
6+	0.051	0.077	0.128	−320.00	0.107	−0.248	−0.141	−352.50
**Wealth (2 categories)**								
Non-richest (ref)								
Richest	0.001	−0.01	−0.008	20.00				
**Wealth quintile**								
Poorest (ref)								
Poorer					−0.003	0.083	0.08	200.00
Middle					0.035	0.04	0.075	187.50
Richer					−0.006	−0.326	−0.332	−830.00
Richest					−0.052	−0.559	−0.611	−1527.50
**ECI2016-ECI2011**			**−0.04**				**0.04**	

ECI2016-ECI2011 refers to the difference between the ECI of 2016 and 2011. ECI refers to the Erreygers concentration index.

**Table 4:** Factors contributing to the change in educational disparities in ANC4+, 2011–2016 EDHS.

**Variables**	**Urban**				**Rural**			
	**Change in CI**	**Change in elasticity**	**Total change**	**%**	**Change in CI**	**Change in elasticity**	**Total change**	**%**
**Age at pregnancy**								
Not adolescent (ref)								
Adolescent	0.001	0.001	0.002	−1.18	−0.026	0.049	0.023	57.50
**Region**								
Tigray (ref)								
Afar	0.003	−0.001	0.003	−1.76	−0.002	0.002	0	0.00
Amhara	−0.046	−0.061	−0.107	62.94	−0.071	−0.08	−0.15	−375.00
Oromia	−0.01	−0.006	−0.016	9.41	0.246	−0.129	0.117	292.50
Somali	0.004	−0.041	−0.037	21.76	−0.016	0.056	0.039	97.50
Benisahangul Gumuz	−0.001	−0.003	−0.003	1.76	−0.003	0.001	−0.002	−5.00
SNNP	0.051	−0.014	0.037	−21.76	−0.033	−0.016	−0.049	−122.50
Gambela	−0.001	0	−0.001	0.59	0	−0.006	−0.006	−15.00
Harar	0.003	−0.003	0.001	−0.59	0	0	0	0.00
Addis Ababa	−0.002	−0.068	−0.07	41.18	
Dire Dawa	0.003	0	0.003	−1.76	0	−0.001	0	0.00
**Partner's education**								
No education (ref)								
Primary	−0.078	0.019	−0.059	34.71	−0.01	−0.02	−0.03	−75.00
Secondary	−0.01	−0.001	−0.011	6.47	0.001	−0.001	0	0.00
Higher	−0.063	−0.055	−0.118	69.41	−0.005	−0.013	−0.018	−45.00
**Religion**								
Orthodox (ref)								
Protestant	0.006	0.028	0.034	−20.00	−0.009	−0.04	−0.05	−125.00
Muslim	0.002	−0.014	−0.012	7.06	−0.004	−0.014	−0.018	−45.00
Other	−0.005	−0.003	−0.008	4.71	0.01	0.006	0.016	40.00
**Media exposure**								
Not exposed (ref)								
Exposed	0.002	−0.001	0	0.00	0.005	−0.081	−0.076	−190.00
**Women's education**								
No education (ref)								
Primary	0.003	0.096	0.099	−58.24	−0.039	−0.308	−0.347	−867.50
Secondary/higher	−0.021	−0.474	−0.495	291.18	−0.013	−0.012	−0.025	−62.50
**Women's occupation**								
Has no occupation (ref)								
Has occupation	0	0.001	0.001	−0.59	0	−0.027	−0.027	−67.50
**Partner's occupation**								
Has no occupation (ref)								
Has occupation	0	0.031	0.031	−18.24	0.006	−0.001	0.005	12.50
**Attitude toward violence**								
Low (ref)								
Medium	0.025	−0.005	0.02	−11.76	0.003	−0.019	−0.016	−40.00
High	−0.028	0.015	−0.014	8.24	0.002	−0.071	−0.068	−170.00
**Social independence**								
Low (ref)								
Medium	0.013	0.003	0.015	−8.82	−0.002	0.004	0.002	5.00
High	−0.014	0.063	0.049	−28.82	0.001	0.064	0.065	162.50
**Decision-making**								
Low (ref)								
Medium	0.006	−0.039	−0.032	18.82	−0.005	−0.004	−0.009	−22.50
High	−0.034	0.057	0.023	−13.53	−0.004	−0.045	−0.049	−122.50
**Birth order**								
1 (ref)								
2-3	0	−0.012	−0.012	7.06	−0.003	0.018	0.015	37.50
4-5	−0.008	−0.026	−0.034	20.00	0.018	0.005	0.024	60.00
6+	−0.009	−0.058	−0.067	39.41	0.041	−0.071	−0.03	−75.00
**Wealth (2 categories)**								
Non-richest (ref)								
Richest	−0.004	0.033	0.03	−17.65				
**Wealth quintile**								
Poorest (ref)								
Poorer					−0.003	0.007	0.003	7.50
Middle					0.03	0.015	0.045	112.50
Richer					−0.008	−0.092	−0.1	−250.00
Richest					−0.045	−0.152	−0.198	−495.00
**ECI2016-ECI2011**			**−0.17**				**0.04**	

ECI2016-ECI2011 refers to the difference between the ECI of 2016 and 2011. ECI refers to the Erreygers concentration index.

**Table 5:** Factors contributing to the change in educational disparities in SBA, 2011–2016 EDHS.

**Variables**	**Urban**				**Rural**			
	**Change in CI**	**Change in elasticity**	**Total change**	**%**	**Change in CI**	**Change in elasticity**	**Total change**	**%**
**Age at birth**								
Not adolescent (ref)								
Adolescent	0	−0.013	−0.013	10.83	−0.007	0.517	0.51	392.31
**Region**								
Tigray (ref)								
Afar	0.014	0.003	0.016	−13.33	−0.002	−0.013	−0.015	−11.54
Amhara	−0.051	0.132	0.081	−67.50	−0.043	0.415	0.373	286.92
Oromia	0.003	−0.039	−0.037	30.83	0.174	−0.284	−0.11	−84.62
Somali	0.032	0.094	0.127	−105.83	0.012	0.112	0.125	96.15
Benisahangul Gumuz	−0.003	0.007	0.004	−3.33	−0.012	0.011	−0.001	−0.77
SNNP	0.111	−0.117	−0.007	5.83	−0.034	−0.027	−0.061	−46.92
Gambela	−0.002	−0.003	−0.005	4.17	0	−0.042	−0.042	−32.31
Harar	0.001	−0.005	−0.004	3.33	−0.001	0.001	0.001	0.77
Addis Ababa	0	−0.097	−0.096	80.00	
Dire Dawa	0.002	0.006	0.008	−6.67	−0.001	0.011	0.01	7.69
**Partner's education**								
No education (ref)								
Primary	0.003	0.04	0.043	−35.83	0.006	−0.275	−0.269	−206.92
Secondary	0.004	−0.018	−0.014	11.67	0.018	0.002	0.02	15.38
Higher	−0.056	0.157	0.101	−84.17	−0.015	0.197	0.182	140.00
**Religion**								
Orthodox (ref)								
Protestant	0.019	0.025	0.045	−37.50	−0.013	0.1	0.087	66.92
Muslim	−0.006	−0.023	−0.029	24.17	−0.001	−0.181	−0.181	−139.23
Other	−0.006	−0.001	−0.006	5.00	0.013	−0.025	−0.012	−9.23
**Media exposure**								
Not exposed (ref)								
Exposed	0.001	−0.014	−0.013	10.83	0.008	−0.011	−0.003	−2.31
**Women's education**								
No education (ref)								
Primary	−0.026	−0.069	−0.095	79.17	−0.03	−0.799	−0.828	−636.92
Secondary/higher	−0.049	−0.127	−0.176	146.67	−0.002	−0.399	−0.402	−309.23
**Women's occupation**								
Has no occupation (ref)								
Has occupation	−0.005	−0.007	−0.011	9.17	0	−0.027	−0.027	−20.77
**Partner's occupation**								
Has no occupation (ref)								
Has occupation	−0.01	−0.014	−0.025	20.83	−0.007	−0.002	−0.009	−6.92
**Attitude toward violence**								
Low (ref)								
Medium	0.003	0.01	0.013	−10.83	0	0.002	0.002	1.54
High	0.006	−0.166	−0.16	133.33	−0.02	0.032	0.012	9.23
**Social independence**								
Low (ref)								
Medium	−0.011	−0.021	−0.032	26.67	0	0.178	0.179	137.69
High	−0.031	0.062	0.03	−25.00	−0.008	0.197	0.19	146.15
**Decision-making**								
Low (ref)								
Medium	−0.009	0.037	0.028	−23.33	−0.003	−0.024	−0.026	−20.00
High	0.006	−0.042	−0.037	30.83	0.001	−0.107	−0.106	−81.54
**Birth order**								
1 (ref)								
2-3	−0.007	−0.015	−0.022	18.33	−0.028	0.16	0.132	101.54
4-5	0.03	0.045	0.075	−62.50	0.036	−0.508	−0.472	−363.08
6+	0.037	0.003	0.039	−32.50	0.15	−0.591	−0.441	−339.23
**Wealth (2 categories)**								
Non-richest (ref)								
Richest	0.003	−0.08	−0.077	64.17				
**Wealth quintile**								
Poorest (ref)								
Poorer					0.028	0.119	0.147	113.08
Middle					0.046	0.033	0.079	60.77
Richer					−0.037	−0.735	−0.772	−593.85
Richest					−0.028	−0.736	−0.764	−587.69
**ECI2016-ECI2011**			**−0.12**				**0.13**	

ECI2016-ECI2011 refers to the difference between the ECI of 2016 and 2011. ECI refers to the Erreygers concentration index.

**Table 6:** Factors contributing to the change in educational disparities in PNC, 2011–2016 EDHS.

**Variables**	**Urban**				**Rural**		
	**Change in CI**	**Change in elasticity**	**Total change**	**%**	**Change in CI**	**Change in elasticity**	**Total change**	**%**
**Age at pregnancy**								
Not adolescent (ref)								
Adolescent	0.004	0	0.004	−2.67	0.009	1.418	1.427	1,783.75
**Region**								
Tigray (ref)								
Afar	0.005	0.005	0.01	−6.67	−0.003	−0.106	−0.11	−137.50
Amhara	−0.024	−0.02	−0.045	30.00	−0.164	−0.576	−0.739	−923.75
Oromia	−0.008	−0.003	−0.011	7.33	0.556	0.036	0.592	740.00
Somali	0.003	−0.007	−0.005	3.33	−0.017	−0.025	−0.042	−52.50
Benisahangul Gumuz	−0.004	0.008	0.004	−2.67	−0.012	−0.012	−0.024	−30.00
SNNP	0.047	−0.039	0.008	−5.33	−0.093	0.435	0.342	427.50
Gambela	−0.001	0	−0.002	1.33	0.001	−0.049	−0.049	−61.25
Harar	0	−0.002	−0.002	1.33	0	0.007	0.007	8.75
Addis Ababa	0.005	−0.046	−0.041	27.33	
Dire Dawa	0.005	−0.001	0.005	−3.33	−0.001	0.009	0.007	8.75
**Partner's education**								
No education (ref)								
Primary	−0.02	0.025	0.005	−3.33	0	0.471	0.471	588.75
Secondary	0.006	−0.184	−0.178	118.67	0	0.251	0.251	313.75
Higher	0.036	−0.541	−0.505	336.67	−0.042	1.151	1.108	1385.00
**Religion**								
Orthodox (ref)								
Protestant	−0.022	0.036	0.014	−9.33	−0.018	−0.243	−0.261	−326.25
Muslim	0	−0.022	−0.022	14.67	0.002	−0.437	−0.435	−543.75
Other	0.006	−0.004	0.002	−1.33	0.046	0.027	0.073	91.25
**Media exposure**								
Not exposed (ref)								
Exposed	−0.003	−0.02	−0.023	15.33	0.026	0.47	0.496	620.00
**Women's education**								
No education (ref)								
Primary	−0.021	0.048	0.027	−18.00	−0.091	−1.342	−1.434	−1792.50
Secondary/higher	−0.115	−0.119	−0.233	155.33	−0.014	−4.429	−4.443	−5553.75
**Women's occupation**								
Has no occupation (ref)								
Has occupation	0.001	−0.003	−0.002	1.33	0	−0.037	−0.037	−46.25
**Partner's occupation**								
Has no occupation (ref)								
Has occupation	−0.001	−0.006	−0.006	4.00	0.016	0.15	0.167	208.75
**Attitude toward violence**								
Low (ref)								
Medium	0.007	0.01	0.017	−11.33	0.002	0.157	0.16	200.00
High	0.002	−0.04	−0.038	25.33	0.023	−0.694	−0.671	−838.75
**Social independence**								
Low (ref)								
Medium	0.002	0.007	0.009	−6.00	0.011	1.276	1.287	1608.75
High	0.005	−0.011	−0.006	4.00	0.006	0.475	0.481	601.25
**Decision-making**								
Low (ref)								
Medium	0.024	−0.11	−0.086	57.33	−0.016	−0.004	−0.019	−23.75
High	−0.098	0.291	0.193	−128.67	−0.006	0.135	0.129	161.25
**Birth order**								
1 (ref)								
2-3	−0.009	0.032	0.023	−15.33	0.001	1.042	1.043	1303.75
4-5	−0.011	−0.043	−0.054	36.00	−0.03	−1.082	−1.112	−1390.00
6+	−0.038	−0.031	−0.069	46.00	0.008	−2.683	−2.675	−3343.75
**Wealth (2 categories)**								
Non-richest (ref)								
Richest	−0.002	−0.124	−0.126	84.00				
**Wealth quintile**								
Poorest (ref)								
Poorer					−0.008	−0.947	−0.955	−1193.75
Middle					0.081	−0.335	−0.254	−317.50
Richer					−0.011	0.25	0.239	298.75
Richest					−0.167	0.233	0.067	83.75
**ECI2016-ECI2011**			**−0.15**				**0.08**	

ECI2016-ECI2011 refers to the difference between the ECI of 2016 and 2011. ECI refers to the Erreygers concentration index.

### Contributions to the change in disparities in maternal health care services in urban settings

Contributions of the factors to the change in the disparities (Tables 2–6) varied in direction and size based on the type of health care services. The educational status of women, mainly secondary or higher education, is one of the most important factors and contributes to the narrowing or widening of the disparities. For instance, it substantially contributes (373.64%) to the narrowing of the inequality in ANC quality. This is mainly due to the change in direction and the reduction in the magnitude of its elasticity over time, from 0.373 in 2011 to −0.341 in 2016. However, it impacted the fall in disparity in early ANC differently; specifically, it contributed −1,075% to the gap, with the negative sign indicating that contribution widened the gap. The change in its elasticity from negative (−0.647) in 2011 to positive (0.02) in 2016 accounted for a larger portion of its contribution to the disparity. See Tables 2–6 for the details.

### Contributions to the change in disparities in maternal health care services in rural settings

The factors that contributed to the disparities in urban areas also had a role in the disparities in rural settings. Similar to the case of disparities in urban places, the factors' contributions to the shift in the disparities varied in sign and magnitude by the type of health care services. The percentage contributions are, for most of the factors, very large because the change in disparities in the maternal health care services (used as the denominator for the percentage calculations) in rural settings are either smaller than that of urban areas or the changes in elasticities and concertation indices of the factors are more noticeable in rural areas than in urban areas. Maternal education, women empowerment, partner education, region, wealth, birth order, and age are among the variables that contribute to the change in the gap the most. For example, women's education contributed −930% to the change in the disparity in ANC4+, with most of the contribution coming from primary education. The influence of primary education was largely driven by a change in its elasticity (−0.308), with a secondary impact from a change in its concentration index (−0.039). The richest wealth quintile made one of the highest contributions (−587.69%) to the change in the disparity in SBA, with the majority of this contribution stemming from a change in its elasticity (−0.736). See Tables 2–6 for the details.

## Discussion

The study attempted to examine whether educational disparities in maternal health care services were altered over time using relative and absolute measures of inequality and to suggest mechanisms that potentially drive the change of the disparities. The study unveiled that all five maternal health care services exhibited a “*pro-educated”* type of distribution, where the services were distributed unequally and favored women with more education in both surveys. The education-related disparities evolved into a different state and were patterned differently for urban and rural areas. Interestingly, the disparities were narrowed in urban areas, but in rural areas, while the relative inequalities suggested conflicting conclusions, the absolute disparities worsened over time. Despite a drop in urban absolute disparities, the inequalities in rural areas were less pronounced than in urban areas. Finally, the analysis showed that the change in absolute inequalities did not appear out of a vacuum; instead, many factors, including maternal education, emerged as the potential drivers of the alterations in the disparities.

The contribution of determinants to inequality in an outcome variable is a function of their elasticities and concentration index. Factors that had strong associations with the outcome, as reflected by their higher elasticities, are unequally distributed across the inequality ranking variable (e.g., education in this study), as reflected by their higher concentration index, and contribute more to the inequality. Our analyses revealed that maternal education played an essential role in the change of education-related disparities, and its contribution to the change is mainly linked to the change in its nexus with professional maternal health care. Maternal education is among the significant social determinants and is related to the utilization of skilled maternal health care (30, 44). In this study, maternal education contributes considerably to the change in disparities. The finding suggests that equity in maternal health care services can be improved by increasing secondary or higher female education coverage. While Ethiopia has made great strides in student enrollment, advancement into secondary education is deficient—only about 33% of students join secondary education (45). The SDGs are deemed related—often, fulfilling one SDG requires addressing challenges related to another. Education for girls and equality between men and women is critical if the world is to meet all of the SDGs, including SDG 3 (29). Some of the other major drivers of the change included partner education, empowerment of women, and birth order, all of which are linked with the uptake of maternal health care services (30, 46).

The fall of the disparities in urban settings may be related to the alteration in behaviors of the determinants in reaction to some reasons, such as policy change, change in knowledge on the importance of professional health services, and expansion of health facilities. The analyses showed that influences on the changes of the disparities of some determinants stem mainly from the drop in their elasticities in the second period, suggesting that women in different categories of these determinants had become relatively more equal in terms of getting the services in 2016 than in 2011. Since a change of elasticity measures a change in responsiveness of the services to changes in the factors, a reduction of elasticities may suggest that utilization of maternal health care services might have been improved over time. Over the recent years, the expansion of health facilities in Ethiopia increased and might have raised the chance of people's access to maternal health care services (47) and might thus have driven the reduction of disparities in urban places.

It is worth noting that narrowing disparities in maternal health care with changes in elasticities has essential policy ramifications. Increased availability of medical care opportunities may allow many women to access maternal health care, and this increased accessibility of health care over time may substantially improve equities in health care, but this would happen without their socioeconomic realities being addressed. The sheer improvement of equity in health care should not mask the need to significantly improve disparities in social determinants such as in the economy. The other point is that gaps in health care can be reduced when elasticities are changed, yet the reduced gaps may not necessarily point to an improvement in equity. Instead, it may simply suggest a change in the subpopulation that obtains more health care. For example, in this study, the drop in the elasticity of the ANC quality with secondary or higher education of women from 3.73 to −0.341 had a more significant share of the reduction in the ANC quality disparity than the change in its concentration index. Although the drop in elasticity drives reduction significantly, this occurred at the expense of decreased service utilization for more educated women.

The economic problem is an established barrier to accessing professional maternal health care in Africa (48), a region with poor accessibility to health care and a higher burden of diseases. In the event of a lack of income and consumption data, the wealth index is a valid proxy for SES in LMICs (49). Research literature suggests that maternal health care services utilization differs by wealth (50). In this study, the wealthiest quintile was among the drivers that led to the overtime change in the disparities, where its influence came mainly from the change in its elasticity during the same time. The wealth index had complex and varying roles in the inequality of different health care. It contributed to the shrinking of the disparities in early ANC, SBA, and PNC in urban and rural areas but only contributed to reducing the gap in ANC quality and ANC4+ in rural areas. On the other hand, it contributed to widening the disparities in ANC quality and ANC4+ in urban areas. The study indicated that women's economic and educational standings are interconnected and determine inequalities in maternal health care services. In poor and gender-unequal countries such as Ethiopia, women bear a disproportionately higher burden of unemployment and unpaid work (51), and financial barriers to accessing maternal health care must be addressed in different innovative ways (52). Literature suggests that despite officially existing exemption policies, women in LIMCs bear financial burdens and unofficial payments for accessing maternal health care (53). This indicates the need for more commitment to implementing the existing schemes, programs, and policies (54). The story is similar in Ethiopia, where the exemption of maternal health care has been part of the health care policy (55). However, some health facilities still have women paid user fees for delivery services with more significant regional variations and whether health care facilities are public or private (56). In developing countries, poor women lag behind their affluent peers in access to maternal health care even in urban places (57), and much work is needed to abolish the economic disparities in access to maternal health care in both urban and rural sites.

Overall, our study found that, although absolute differences grew with time, there were less noticeable educational disparities in maternal health care services in rural areas than in urban locations. In rural places, due to limited access to health care services, women with lower educational backgrounds may have an even playing field as they experience comparable access to health care services as their more educated counterparts. Conversely, urban areas, with their better health care facilities and services, may witness more pronounced disparities in access based on socioeconomic status. Moreover, the significance of cultural and social norms within rural communities should not be overlooked. Existing evidence shows that maternal health care utilization is shaped by these cultural beliefs and practices (58). In some rural communities, traditional practices and beliefs may play a significant role in shaping women's attitudes toward maternal health care, regardless of their educational background (59). This can create a more level playing field in terms of health care utilization, as education may have less of an impact on women's health care decisions in these communities.

Our finding emphasizes the importance of enacting context-specific policies and strategies to effectively tackle the varying disparities in rural and urban areas. However, undertaking qualitative research could offer valuable insights into the area-specific structural and individual-level factors that drive the differing levels of educational disparities in urban and rural settings.

The study has a few strengths. The application of both absolute and relative, as well as simple and complex measures of disparity, allows for investigating disparities from diverse perspectives. Delving into urban and rural educational disparities through the decomposition analyses helps to expose insights for overcoming inequalities, enabling the development of targeted strategies informed by the contributions of social determinants.

However, the study has a few weaknesses. There were a few observations for some categories for some variables, such as region and religion. Since the EDHS are cross-sectional studies, conclusions drawn from them ought to point toward associations rather than causes. Finally, the data on ANC and PNC services were collected for births that were born last. This restriction to the last birth may limit the relevance of the findings to non-lastborn children, as differences have been recorded between the last-born and non-last-born children (60).

## Conclusion

Both rural and urban areas had educational disparities in maternal health care services. However, the disparities were not as severe in rural areas as they were in urban areas. Urban disparities showed improvement in both absolute and relative measures, whereas rural disparities showed mixed results in the relative measures and a rise in the absolute index. The change in the inequalities of the determinants and how they relate to maternal health care services was linked with the changes in the absolute educational disparities. Social policies in the country should emphasize education advancement, economic development, empowerment of women, job creation, and health education.

## Data availability statement

The EDHS dataset analyzed during the current study is publicly available and can be found on the DHS website: http://www.dhsprogram.com.

## Ethics statement

The surveys were approved by the ICF IRB and IRB in Ethiopia. The studies were conducted in accordance with the local legislation and institutional requirements. Written informed consent for participation was not required from the participants or the participants' legal guardians/next of kin because the current study used secondary data, EDHS data. The surveys simply interviewed women of reproductive age and involved no harm to the participants.

## Author contributions

GS: Conceptualization, Data curation, Formal analysis, Investigation, Methodology, Writing—original draft, Writing—review & editing. WM: Supervision, Writing—review & editing. DH: Supervision, Writing—review & editing.
