# Supplementary material for: Explaining changes in educational disparities in competent maternal health care services in urban and rural areas in Ethiopia
Source: Front Public Health. 2024 Apr 12;12:1332801. doi: 10.3389/fpubh.2024.1332801 (PMC11045905; doi:10.3389/fpubh.2024.1332801)
Supplement: Supplementary file 1 [file Data_Sheet_1.docx]

**Measuring interaction effect in the context of non-linear models**

In non-linear models like logistics and Poisson regressions, an interaction term between, for example, two nominal variables do not necessarily measure an interaction effect. In such context, analysts can simply include the product term in their model to see whether the effect on an outcome of one variable varies by the level of another variable in the model, yet ignore the **coefficient of the product term** interpreting it as the measure of the interaction. Instead, compute marginal effects for each exposure variable, and then, compute the difference between the two marginal effects, called the **second difference** of the two marginal effects. In nonlinear models, marginal effects are essential for interpreting parameters, and the second difference in marginal effects is valuable to assess interaction effects (1).

In our study, we were interested in measuring whether the educational disparity in maternal healthcare services changed over time, and have done interaction between education (no education, primary, secondary, and higher) and survey year (2011 and 2016), controlling for other factors. We calculated average marginal effects (AME) for education (for higher education vs no education) for each year, and then, we took the difference between the AME of 2016 and that of 2011 to get the difference in difference, or **second difference of the AMEs**.

$$Log odd\left( healthcare \right)= a+\beta1education+\beta2survey+\beta3education*survey+\beta4Z$$

Where a is the regression constant, β1 is the effect of education in log-odds metric, β2 is the effect of survey year in log-odds metric, and β4 is the effect of other variables in log-odds metric. As explained above, β3 does not necessarily provide information about the interaction between education and survey year. So, we need to convert the model into the one that has estimates in probability scale. This can easily be done using the mtable Stata command.

**We used Stata codes (below) to do the second differences for disparities in urban areas for the ANC quality outcome variable.** Second differences for the other outcome variables and also for rural disparities are done in the same way.

***For educational disparity in urban areas

svy: logit anc_quality_binary i.adolescent_age i.region i.husband_edu i.religion_f i.media_new i.wealth_rr i.women_occupation i.husband_occupation i.swpatt3gr i.swpsoc3gr i.swpdec3gr i.birthor i.meduc_t00##i.surve16 if v025==1 // **logit model**

The “**svy**” prefix is used to account for the complex behavior of the DHS data. It is used to produce correct point estimates and standard errors. The **svy** usage is related to just the data and has nothing to do with the estimation of AME and second differences.

**mtable**, at(meduc_t00=(0 2)) over(surve16) post// **produce predictive probabilities for education (no education and higher education categories) and each survey year.** The *mtable* Stata command predicts the probability that women use the ANC service instead of not using it for each category of the categorical variables (education and survey year).

*Effect of high vs low education for 2011

mlincom 3 – 1 // **AME**

This command computes the AME of high education on the use of ANC compared to that of no education in 2011.

*Effect of high vs low education for 2016

mlincom 4 - 2 // **AME**

This command computes the AME of high education on the use of ANC compared to that of no education in 2016.

***2nd difference**

mlincom (4 - 2) - (3 - 1)

- This command computes the difference in AME of 2016 and that of 2011. The second difference then measures how the educational disparity changed between 2011 and 2016, which reflects the concept of the changing effect of education on the different values (2011 and 2016) of another variable (survey year), something that interaction is about.

**Oaxaca-type decomposition**

Analyzing whether there are differences between women who are educated and those who are not in terms of healthcare services received is one thing. It is another thing altogether to try and explain why that difference occurred in the first place. The latter is more practically significant since it requires this evidence to close gaps.

Blinder-Oaxaca (BO) decomposition is an approach that has been used in the literature to explain apparent gaps (disparities) by breaking down the differences between two groups. The methodology decomposes the mean difference in outcomes between two groups into the contributions of variables that emerged in the model. The inequality is broken down into two components: one arising from disparities in the magnitudes of the variables impacting the outcome variable among different groups, and the other emanating from variations in how these factors influence the outcome among those groups. For instance, penurious women may experience barriers in accessing maternal healthcare services due to both the limited availability of health infrastructure and their poor agency in traversing the process of getting these maternal healthcare services. In a situation where causal effects are possible to be obtained, the BO decomposition aids in informing the sensitive policy decision process by providing the contribution of variables of interest to the disparity in a simple and natural metric, percent.

Apart from its natural decomposition application on the two-group disparity in one-time dimension, the method can interestingly be extended to estimate the difference between two concentration indices-related disparities computed at two time periods. The procedure is much like difference-in-difference. Using this strategy, one can break down changes in socioeconomic inequality in healthcare or health into two categories: changes in healthcare's or health's elasticity concerning factors and changes in inequality in those factors of health. Practically, it is a two-step procedure in Stata. We first decomposed the educational disparities in 2011 and 2016 separately using the convenient regression method (this is explained sufficiently in the main text in the method section) (2). Then, we applied the **Oaxaca-type decomposition to take the difference between the disparities in 2011 and that of 2016, to get a two-dimensional view of the educational disparity (in terms of time and space).**

**Stata code for Oaxaca-type decomposition for decomposing change in disparity over time.**

*2011 (survey=3) // decompose inequalities for the year 2011

**clear matrix**

conindex anc_quality_binary if survey==3 & v025==1, rankvar(meduc_t00) bounded limits(0 1) erreygers svy **//** **to compute concentration index**

sca EI2011 = r(CI) **// store the index**

**global** X adolescent_age_ur2 region_ur2 region_ur3 region_ur4 region_ur5 region_ur6 region_ur7 region_ur8 region_ur9 region_ur10 region_ur11 husband_edu_ur2 husband_edu_ur3 husband_edu_ur4 religion_f_ur2 religion_f_ur3 religion_f_ur4 media_new_ur2 meduc_t00_ur2 meduc_t00_ur3 women_occupation_ur2 husband_occupation_ur2 swpatt3gr_ur2 swpatt3gr_ur3 swpsoc3gr_ur2 swpsoc3gr_ur3 swpdec3gr_ur2 swpdec3gr_ur3 birthor_ur2 birthor_ur3 birthor_ur4 wealth_rr_ur2 **// global to capture the variables in the model**

qui sum anc_quality_binary if survey==3 & v025==1 [aw=wt] **// summary**

sca m_y=r(mean) **// store the mean of the outcome variable**

svy: qui glm anc_quality_binary $X if survey==3 & v025==1, family(binomial) link(logit) **// regression**

foreach x of varlist $X {

sca b_`x'=_b[`x']

}

foreach x of varlist $X {

qui {

conindex `x' if survey==3 & v025==1, rank(meduc_t00) truezero svy

sca CI2011_`x' = r(CI)

sum `x' if survey==3 & v025==1 [aw=wt]

sca m_`x'=r(mean)

sca elas2011_`x' = (b_`x'*m_`x')/m_y

sca con_`x' = 4*b_`x'*m_`x'*CI2011_`x'

sca prcnt_`x' = 100*con_`x'/EI2011

}

}

* 2016 and the change // **decompose inequalities for the year 2016** **and decompose the change**

** repeat the above step

**clear matrix**

conindex anc_quality_binary if survey==4 & v025==1, rankvar(meduc_t00) bounded limits(0 1) erreygers svy

sca EI2016 = r(CI)

**global X** adolescent_age_ur2 region_ur2 region_ur3 region_ur4 region_ur5 region_ur6 region_ur7 region_ur8 region_ur9 region_ur10 region_ur11 husband_edu_ur2 husband_edu_ur3 husband_edu_ur4 religion_f_ur2 religion_f_ur3 religion_f_ur4 media_new_ur2 meduc_t00_ur2 meduc_t00_ur3 women_occupation_ur2 husband_occupation_ur2 swpatt3gr_ur2 swpatt3gr_ur3 swpsoc3gr_ur2 swpsoc3gr_ur3 swpdec3gr_ur2 swpdec3gr_ur3 birthor_ur2 birthor_ur3 birthor_ur4 wealth_rr_ur2

qui sum anc_quality_binary if survey==4 & v025==1 [aw=wt]

sca m_y=r(mean)

svy: qui glm anc_quality_binary $X if survey==4 & v025==1, family(binomial) link(logit)

foreach x of varlist $X {

sca b_`x'=_b[`x']

}

foreach x of varlist $X {

qui {

conindex `x' if survey==4 & v025==1, rank(meduc_t00) truezero svy

sca CI2016_`x' = r(CI)

sum `x' if survey==4 & v025==1 [aw=wt]

sca m_`x'=r(mean)

sca elas2016_`x' = (b_`x'*m_`x')/m_y

sca con_`x' = 4*b_`x'*m_`x'*CI2016_`x'

sca prcnt_`x' = 100*con_`x'/EI2016

sca che_CI`x' = elas2016_`x'*(CI2016_`x' - CI2011_`x') // **change in concentration index**

sca che_Ela`x' = CI2011_`x'*(elas2016_`x' - elas2011_`x')// **change in elasticity**

sca CC`x' = elas2016_`x'*(CI2016_`x' - CI2011_`x') + CI2011_`x'*(elas2016_`x' - elas2011_`x')

} // total **change**

di "`xí concentration index:", che_CI`x'

di "`xí elast:", che_Ela`x'

di "`xí totalCC:", CC`x'

matrix Decomp20112016_urban = nullmat(Decomp20112016_urban)\ (che_CI`x', che_Ela`x', CC`x')

}

matrix rownames Decomp20112016_urban= $X

matrix colnames Decomp20112016_urban = "CIchange""elaschnage""CC"

matrix list Decomp20112016_urban, format(%8.3f)

esttab matrix(Decomp20112016_urban, fmt(3)) using ANCQdecompostion_Change_noboot.txt, mtitle("") nonum replace lines nonum title(Table ANCQ: Decomposition of change in ANCQ No booting urban 2011-2016) // **export the result**

**SWPER Global index**

The SWPER Global index is a suitable common measure of women’s empowerment for LMICs using DHS data and allows for measuring progress across time and countries at the individual and country levels. The SWPER Global involves three empowerment domains suggestive of assets and agency among married women(3):

1. **Social independence**: mainly captures preconditions that enable women to achieve their goals (schooling attainment, access to information, age at pivotal life events, and spousal asset differentials and access to information).
2. **Decision-making**: measures the degree of a woman’s participation in household decisions, something that reflects instrumental agency.
3. **Attitudes to violence**: this domain is linked to the issue of intrinsic agency and serves as a proxy for women’s acceptability of gender-related violence.

Fourteen variables were used to derive the three domains of the index. So, every time this index is referred to, at least in our paper, the three domains are referred to as standalone categorical indicators. The Principal Components Analyses (PCA) statistical data reduction method was used in survey data to obtain the scores and to identify the three domains of the index. The authors of the index took helpful suggestions from the panel of experts to refine a more localized version of the empowerment indicator previously developed. The index construction process progressed through a series of validity checks to ensure that the index is valid and useful for measuring empowerment. For example, to examine the external validity of the SWPER, Spearman correlation coefficients were computed between the resulting score and the two commonly used indices: the Gender Development Index (GDI) and the Gender Inequality Index (GII). The authors of the index have provided a step-by-step explanation of how they developed the index along with the article they have written, and the article is referred to in the main text of this paper.

**References**

1. Mize T. Best Practices for Estimating, Interpreting, and Presenting Nonlinear Interaction Effects. Sociol Sci [Internet]. 2019 [cited 2023 Jul 18];6:81–117. Available from: https://www.sociologicalscience.com/articles-v6-4-81/.

2. O’Donnell O, van Doorslaer E, Wagstaff A, Lindelow M. Analyzing Health Equity Using Household Survey Data. A Guide to Techniques and Their Implementation [Internet]. Washington: World Bank; 2008[cited 2023 Jul 14]. 234 p.

3. Ewerling F, Raj A, Victora CG, Hellwig F, Coll CV, Barros AJ. SWPER Global: A survey-based women’s empowerment index expanded from Africa to all low- and middle-income countries. J Glob Health[Internet]. 2020[cited 2024 March 6]; 10(2). Available from: pmid:33274055.
