# Supplementary material for: Explaining changes in educational disparities in competent maternal health care services in urban and rural areas in Ethiopia
Source: Front Public Health. 2024 Apr 12;12:1332801. doi: 10.3389/fpubh.2024.1332801 (PMC11045905; doi:10.3389/fpubh.2024.1332801)
Supplement: Supplementary file 2 [file Data_Sheet_2.docx]

**Table 1. Elasticities, concentration indices, and contributions of factors to disparity in ANC quality, 2011 and 2016 EDHS**

|  | **Rural** | | | | | | | | **Urban** | | | | | | | |
| --- | --- | --- | --- | --- | --- | --- | --- | --- | --- | --- | --- | --- | --- | --- | --- | --- |
| **Variables** | **2011** | | | | **2016** | | | | **2011** | | | | **2016** | | | |
|  | **Elasticity** | **CI** | **absolute** | **%** | **Elasticity** | **CI** | **Absolute** | **%** | **Elasticity** | **CI** | **Absolute** | **%** | **Elasticity** | **CI** | **Absolute** | **%** |
| **Age (years)** |  |  |  |  |  |  |  |  |  |  |  |  |  |  |  |  |
| <20 | -0.356 | 0.178 | -0.066 | -62.766 | -0.088 | 0.263 | -0.051 | -45.392 | 0.139 | 0.021 | 0.008 | 3.319 | -0.078 | 0.097 | -0.025 | -20.828 |
| **Region** |  |  |  |  |  |  |  |  |  |  |  |  |  |  |  |  |
| Afar | 0.009 | -0.29 | -0.003 | -2.638 | -0.019 | -0.245 | 0.01 | 9.098 | -0.014 | -0.193 | 0.007 | 3.141 | -0.003 | -0.346 | 0.004 | 3.3 |
| Amhara | -0.992 | -0.091 | 0.094 | 89.611 | -0.327 | -0.036 | 0.026 | 23.443 | -0.383 | -0.11 | 0.11 | 48.335 | -0.115 | -0.009 | 0.004 | 2.9 |
| Oromia | 0.279 | 0.047 | 0.014 | 13.113 | -0.759 | -0.026 | 0.044 | 38.78 | -0.383 | 0.034 | -0.033 | -14.728 | -0.238 | 0.047 | -0.038 | -30.965 |
| Somali | 0.043 | -0.28 | -0.012 | -11.88 | -0.033 | -0.225 | 0.016 | 14.668 | -0.016 | -0.418 | 0.017 | 7.671 | -0.01 | -0.462 | 0.015 | 12.201 |
| B. Gumuz | -0.015 | -0.044 | 0.001 | 0.647 | -0.02 | 0.038 | -0.002 | -1.541 | -0.022 | -0.123 | 0.007 | 3.169 | -0.001 | -0.002 | 0 | 0.006 |
| SNNP | -0.435 | 0.058 | -0.026 | -24.981 | -0.441 | 0.091 | -0.089 | -78.922 | -0.098 | 0.073 | -0.018 | -8.142 | -0.154 | -0.094 | 0.048 | 40.036 |
| Gambela | 0.004 | 0.228 | 0.001 | 1.01 | -0.003 | 0.192 | -0.001 | -1.032 | -0.01 | 0.013 | 0 | -0.141 | -0.006 | 0.065 | -0.001 | -1.104 |
| Harar | 0.008 | -0.085 | -0.001 | -0.702 | -0.001 | -0.055 | 0 | 0.064 | 0.019 | 0.14 | 0.007 | 3.106 | 0.006 | -0.051 | -0.001 | -0.881 |
| Addis Ababa |  |  |  |  |  |  |  |  | 0.765 | 0.112 | 0.224 | 98.551 | 0.12 | 0.07 | 0.028 | 23.43 |
| Dire Dawa | 0.004 | -0.165 | -0.001 | -0.586 | 0 | -0.082 | 0 | -0.015 | 0.026 | 0.017 | 0.001 | 0.506 | 0.009 | -0.125 | -0.004 | -3.133 |
| **Partner education** |  |  |  |  |  |  |  |  |  |  |  |  |  |  |  |  |
| Primary | -0.513 | 0.137 | -0.073 | -69.806 | 0.088 | 0.105 | 0.02 | 18.042 | 0.509 | -0.113 | -0.15 | -66.237 | 0.406 | -0.261 | -0.355 | -293.393 |
| Secondary | -0.022 | 0.472 | -0.011 | -10.404 | 0.053 | 0.476 | 0.055 | 49.4 | 0.45 | 0.212 | 0.249 | 109.668 | 0.336 | 0.179 | 0.202 | 166.613 |
| Higher | 0.04 | 0.774 | 0.032 | 31.045 | 0.012 | 0.591 | 0.016 | 13.876 | 0.321 | 0.52 | 0.434 | 191.388 | 0.544 | 0.374 | 0.682 | 563.263 |
| **Religion** |  |  |  |  |  |  |  |  |  |  |  |  |  |  |  |  |
| Protestant | -0.626 | 0.092 | -0.059 | -56.954 | -0.255 | 0.112 | -0.063 | -56.27 | -0.029 | 0.081 | -0.006 | -2.666 | -0.153 | -0.031 | 0.016 | 12.931 |
| Muslim | -0.305 | -0.052 | 0.016 | 15.606 | 0.143 | -0.067 | -0.021 | -18.712 | -0.105 | -0.138 | 0.038 | 16.652 | -0.038 | -0.174 | 0.022 | 18.326 |
| Other | -0.065 | -0.06 | 0.004 | 3.901 | 0.01 | -0.161 | -0.004 | -3.151 | -0.057 | -0.072 | 0.011 | 4.679 | -0.023 | 0.285 | -0.022 | -17.903 |
| **Media exposure** |  |  |  |  |  |  |  |  |  |  |  |  |  |  |  |  |
| Yes | 0.244 | 0.213 | 0.054 | 51.598 | 0.037 | 0.255 | 0.021 | 18.431 | 0.081 | 0.118 | 0.025 | 10.963 | -0.235 | 0.111 | -0.087 | -72.033 |
| **Women education** |  |  |  |  |  |  |  |  |  |  |  |  |  |  |  |  |
| Primary | 0.641 | 0.562 | 0.373 | 357.288 | 0.058 | 0.491 | 0.063 | 56.342 | 0.574 | -0.129 | -0.193 | -85.181 | -0.095 | -0.341 | 0.108 | 89.443 |
| Secondary/higher | 0.281 | 0.964 | 0.28 | 268.595 | 0.133 | 0.923 | 0.27 | 240.881 | 0.373 | 0.648 | 0.629 | 277.189 | -0.341 | 0.495 | -0.567 | -467.997 |
| **Women occupation** |  |  |  |  |  |  |  |  |  |  |  |  |  |  |  |  |
| Yes | -0.187 | 0.01 | -0.002 | -1.829 | 0.027 | 0.007 | 0 | 0.357 | 0 | -0.007 | 0 | 0.001 | -0.275 | -0.004 | 0.004 | 2.946 |
| **Partner occupation** |  |  |  |  |  |  |  |  |  |  |  |  |  |  |  |  |
| Yes | -5.043 | -0.001 | 0.006 | 5.476 | -0.135 | 0.004 | -0.001 | -1.007 | 0.725 | 0.006 | 0.011 | 4.688 | 0.248 | 0.006 | 0.005 | 4.275 |
| **Attitude toward violence** |  |  |  |  |  |  |  |  |  |  |  |  |  |  |  |  |
| Medium | 0.119 | 0.025 | 0.003 | 2.97 | -0.085 | 0.015 | -0.003 | -2.476 | 0.12 | -0.081 | -0.025 | -11.145 | 0.037 | 0.013 | 0.002 | 1.358 |
| High | 0.28 | 0.094 | 0.027 | 26.191 | -0.056 | 0.061 | -0.007 | -6.641 | 0.524 | 0.158 | 0.215 | 94.783 | 0.225 | 0.1 | 0.075 | 62.046 |
| **Social independence** |  |  |  |  |  |  |  |  |  |  |  |  |  |  |  |  |
| Medium | -0.245 | 0.139 | -0.035 | -33.768 | 0.105 | 0.158 | 0.037 | 32.759 | 0.15 | 0.061 | 0.024 | 10.448 | 0.067 | 0.002 | 0.001 | 0.433 |
| High | -0.318 | 0.323 | -0.106 | -101.872 | 0.007 | 0.346 | 0.005 | 4.678 | -0.027 | 0.467 | -0.033 | -14.666 | 0.457 | 0.383 | 0.587 | 484.396 |
| **Decision making** |  |  |  |  |  |  |  |  |  |  |  |  |  |  |  |  |
| Medium | 0.284 | 0.002 | 0.001 | 0.53 | 0.029 | -0.016 | -0.001 | -0.934 | 0.076 | -0.136 | -0.027 | -11.785 | 0.059 | -0.064 | -0.013 | -10.528 |
| High | 0.223 | 0.016 | 0.004 | 3.651 | -0.079 | 0.012 | -0.002 | -1.864 | 0.274 | 0.071 | 0.051 | 22.297 | 1.01 | 0.029 | 0.097 | 80.363 |
| **Birth order** |  |  |  |  |  |  |  |  |  |  |  |  |  |  |  |  |
| 2-3 | -0.503 | 0.052 | -0.027 | -25.808 | -0.149 | 0.059 | -0.019 | -17.233 | -0.219 | 0.07 | -0.04 | -17.692 | 0.034 | 0.057 | 0.007 | 5.38 |
| 4-5 | -0.076 | -0.045 | 0.004 | 3.402 | -0.233 | -0.089 | 0.046 | 40.64 | 0.099 | -0.159 | -0.041 | -17.933 | -0.054 | -0.189 | 0.034 | 28.304 |
| 6+ | -0.557 | -0.091 | 0.052 | 50.077 | -0.224 | -0.132 | 0.065 | 58.179 | 0.119 | -0.336 | -0.104 | -45.772 | -0.044 | -0.519 | 0.076 | 62.927 |
| **Wealth index** |  |  |  |  |  |  |  |  |  |  |  |  |  |  |  |  |
| Poorer | 0.065 | -0.063 | -0.004 | -4.08 | 0.068 | -0.069 | -0.01 | -9.143 |  |  |  |  |  |  |  |  |
| Middle | 0.452 | -0.024 | -0.011 | -10.605 | 0.105 | 0.016 | 0.004 | 3.414 |  |  |  |  |  |  |  |  |
| Richer | 0.547 | 0.139 | 0.079 | 75.583 | 0.18 | 0.133 | 0.053 | 47.108 |  |  |  |  |  |  |  |  |
| Richest | 0.081 | 0.359 | 0.03 | 28.662 | 0.104 | 0.23 | 0.053 | 46.917 | -0.383 | 0.042 | -0.042 | -18.643 | 0.779 | 0.04 | 0.105 | 86.908 |

***ANC Antenatal care**

**Table 2. Elasticities, concentration indices, and contributions of factors to disparity in early ANC, 2011 and 2016 EDHS**

|  | **Rural** | | | | | | | | **Urban** | | | | | | | |
| --- | --- | --- | --- | --- | --- | --- | --- | --- | --- | --- | --- | --- | --- | --- | --- | --- |
|  | **2011** | | | | **2016** | | | | **2011** | | | | **2016** | | | |
| **Variables** | **Elasticity** | **CI** | **Absolute** | % | **Elasticity** | **CI** | **Absolute** | **%** | **Elasticity** | **CI** | **Absolute** | **%** | **Elasticity** | **CI** | **Absolute** | **%** |
| **Age (years)** |  |  |  |  |  |  |  |  |  |  |  |  |  |  |  |  |
| <20 | -1.643 | 0.178 | -0.062 | -153.762 | -0.345 | 0.263 | -0.047 | -61.719 | 0.318 | 0.021 | 0.008 | 5.117 | -0.041 | 0.097 | -0.007 | -5.678 |
| **Region** |  |  |  |  |  |  |  |  |  |  |  |  |  |  |  |  |
| Afar | 0.123 | -0.29 | -0.008 | -18.874 | -0.032 | -0.245 | 0.004 | 5.345 | 0.019 | -0.193 | -0.005 | -2.877 | 0.013 | -0.346 | -0.008 | -6.246 |
| Amhara | -1.35 | -0.091 | 0.026 | 64.785 | -0.637 | -0.036 | 0.012 | 15.777 | 0.038 | -0.11 | -0.005 | -3.263 | 0.026 | -0.009 | 0 | -0.34 |
| Oromia | -1.177 | 0.047 | -0.012 | -29.415 | -3.765 | -0.026 | 0.05 | 66.404 | 0.218 | 0.034 | 0.009 | 5.65 | -0.345 | 0.047 | -0.028 | -22.93 |
| Somali | 0.105 | -0.28 | -0.006 | -15.516 | -0.19 | -0.225 | 0.022 | 29.106 | 0.042 | -0.418 | -0.022 | -13.464 | -0.006 | -0.462 | 0.005 | 4.204 |
| B. Gumuz | -0.073 | -0.044 | 0.001 | 1.717 | -0.113 | 0.038 | -0.002 | -2.926 | -0.004 | -0.123 | 0.001 | 0.363 | -0.015 | -0.002 | 0 | 0.033 |
| SNNP | -0.463 | 0.058 | -0.006 | -14.134 | -2.037 | 0.091 | -0.095 | -125.848 | 0.551 | 0.073 | 0.05 | 30.936 | -0.069 | -0.094 | 0.011 | 9.184 |
| Gambela | 0.081 | 0.228 | 0.004 | 9.745 | -0.001 | 0.192 | 0 | -0.165 | 0.033 | 0.013 | 0.001 | 0.33 | 0.003 | 0.065 | 0 | 0.286 |
| Harar | 0.017 | -0.085 | 0 | -0.771 | 0.001 | -0.055 | 0 | -0.038 | 0.043 | 0.14 | 0.007 | 4.617 | 0.012 | -0.051 | -0.001 | -0.876 |
| Addis Ababa |  |  |  |  |  |  |  |  | 0.725 | 0.112 | 0.101 | 62.882 | 0.272 | 0.07 | 0.034 | 27.093 |
| Dire Dawa | 0.018 | -0.165 | -0.001 | -1.556 | 0.016 | -0.082 | -0.001 | -0.897 | 0.067 | 0.017 | 0.001 | 0.885 | 0.054 | -0.125 | -0.012 | -9.605 |
| **Partner education** |  |  |  |  |  |  |  |  |  |  |  |  |  |  |  |  |
| Primary | 0.542 | 0.137 | 0.016 | 39.162 | 0.178 | 0.105 | 0.01 | 12.671 | 0.882 | -0.113 | -0.124 | -77.292 | 0.105 | -0.261 | -0.048 | -38.633 |
| Secondary | 0.291 | 0.472 | 0.029 | 72.262 | -0.096 | 0.476 | -0.023 | -30.946 | 0.61 | 0.212 | 0.16 | 99.989 | 0.221 | 0.179 | 0.07 | 56.014 |
| Higher | -0.256 | 0.774 | -0.042 | -104.487 | 0.007 | 0.591 | 0.002 | 2.939 | 0.804 | 0.52 | 0.517 | 322.4 | 0.378 | 0.374 | 0.249 | 199.956 |
| **Religion** |  |  |  |  |  |  |  |  |  |  |  |  |  |  |  |  |
| Protestant | -3.017 | 0.092 | -0.059 | -145.808 | -0.509 | 0.112 | -0.029 | -38.815 | -0.769 | 0.081 | -0.077 | -47.78 | -0.667 | -0.031 | 0.036 | 28.876 |
| Muslim | -2.812 | -0.052 | 0.031 | 76.543 | 0.636 | -0.067 | -0.022 | -28.796 | -0.356 | -0.138 | 0.061 | 37.959 | -0.278 | -0.174 | 0.085 | 68.341 |
| Other | -0.274 | -0.06 | 0.004 | 8.732 | -0.053 | -0.161 | 0.004 | 5.85 | -0.031 | -0.072 | 0.003 | 1.736 | -0.02 | 0.285 | -0.01 | -7.878 |
| **Media exposure** |  |  |  |  |  |  |  |  |  |  |  |  |  |  |  |  |
| Yes | 1.063 | 0.213 | 0.048 | 119.319 | 0.301 | 0.255 | 0.04 | 52.114 | 0.468 | 0.118 | 0.068 | 42.498 | -0.396 | 0.111 | -0.077 | -61.968 |
| **Women education** |  |  |  |  |  |  |  |  |  |  |  |  |  |  |  |  |
| Primary | 2.029 | 0.562 | 0.241 | 600.483 | 1.454 | 0.491 | 0.368 | 485.04 | 0.099 | -0.129 | -0.016 | -9.846 | 0.191 | -0.341 | -0.115 | -92.332 |
| Secondary/higher | 0.974 | 0.964 | 0.198 | 494.482 | 0.415 | 0.923 | 0.198 | 260.764 | -0.647 | 0.648 | -0.519 | -323.893 | 0.02 | 0.495 | 0.018 | 14.357 |
| **Women occupation** |  |  |  |  |  |  |  |  |  |  |  |  |  |  |  |  |
| Yes | 4.872 | 0.01 | 0.01 | 25.333 | 1.578 | 0.007 | 0.006 | 7.287 | -0.018 | -0.007 | 0 | 0.093 | -0.447 | -0.004 | 0.003 | 2.445 |
| **Partner occupation** |  |  |  |  |  |  |  |  |  |  |  |  |  |  |  |  |
| Yes | -7.31 | -0.001 | 0.002 | 4.217 | -1.618 | 0.004 | -0.003 | -4.175 | -6.394 | 0.006 | -0.045 | -27.804 | -0.518 | 0.006 | -0.006 | -4.559 |
| **Attitude toward violence** |  |  |  |  |  |  |  |  |  |  |  |  |  |  |  |  |
| Medium | 0.647 | 0.025 | 0.003 | 8.608 | 0.007 | 0.015 | 0 | 0.069 | 0.629 | -0.081 | -0.063 | -39.478 | 0.236 | 0.013 | 0.005 | 4.38 |
| High | 2.059 | 0.094 | 0.041 | 102.313 | 0.104 | 0.061 | 0.003 | 4.281 | 1.643 | 0.158 | 0.321 | 200.1 | 0.115 | 0.1 | 0.02 | 16.279 |
| **Social independence** |  |  |  |  |  |  |  |  |  |  |  |  |  |  |  |  |
| Medium | -0.4 | 0.139 | -0.012 | -29.341 | -0.416 | 0.158 | -0.034 | -44.775 | -0.421 | 0.061 | -0.032 | -19.705 | -0.157 | 0.002 | -0.001 | -0.521 |
| High | -0.696 | 0.323 | -0.048 | -118.434 | -0.017 | 0.346 | -0.003 | -4.077 | 0.907 | 0.467 | 0.524 | 326.87 | 0.372 | 0.383 | 0.25 | 201.236 |
| **Decision making** |  |  |  |  |  |  |  |  |  |  |  |  |  |  |  |  |
| Medium | -0.504 | 0.002 | 0 | -0.5 | 0.514 | -0.016 | -0.004 | -5.666 | 0.802 | -0.136 | -0.135 | -84.066 | 0.193 | -0.064 | -0.022 | -17.588 |
| High | -0.721 | 0.016 | -0.003 | -6.265 | 1.716 | 0.012 | 0.011 | 14.042 | 2.242 | 0.071 | 0.196 | 122.523 | 1.253 | 0.029 | 0.063 | 50.905 |
| **Birth order** |  |  |  |  |  |  |  |  |  |  |  |  |  |  |  |  |
| 2-3 | -3.87 | 0.052 | -0.042 | -105.466 | -0.782 | 0.059 | -0.024 | -31.316 | 0.593 | 0.07 | 0.052 | 32.241 | -0.394 | 0.057 | -0.039 | -31.621 |
| 4-5 | -1.687 | -0.045 | 0.016 | 39.978 | -0.923 | -0.089 | 0.042 | 55.643 | 0.35 | -0.159 | -0.069 | -42.905 | -0.114 | -0.189 | 0.038 | 30.558 |
| 6+ | -5.329 | -0.091 | 0.102 | 254.549 | -2.597 | -0.132 | 0.176 | 232.584 | -0.051 | -0.336 | 0.021 | 13.195 | -0.279 | -0.519 | 0.255 | 205.025 |
| **Wealth index** |  |  |  |  |  |  |  |  |  |  |  |  |  |  |  |  |
| Poorer | 1.859 | -0.063 | -0.025 | -61.954 | 0.542 | -0.069 | -0.019 | -25.329 |  |  |  |  |  |  |  |  |
| Middle | 2.57 | -0.024 | -0.013 | -32.013 | 0.863 | 0.016 | 0.007 | 9.656 |  |  |  |  |  |  |  |  |
| Richer | 3.357 | 0.139 | 0.099 | 246.631 | 1.02 | 0.133 | 0.07 | 92.405 |  |  |  |  |  |  |  |  |
| Richest | 1.961 | 0.359 | 0.149 | 370.956 | 0.403 | 0.23 | 0.048 | 62.912 | -0.295 | 0.042 | -0.015 | -9.636 | -0.52 | 0.04 | -0.037 | -29.661 |

***ANC Antenatal care**

**Table 3. Elasticities, concentration indices, and contributions of factors to disparity in ANC4+, 2011 and 2016 EDHS**

|  | **Rural** | | | | | | | | **Urban** | | | | | | | |
| --- | --- | --- | --- | --- | --- | --- | --- | --- | --- | --- | --- | --- | --- | --- | --- | --- |
|  | **2011** | | | | **2016** | | | | **2011** | | | | **2016** | | | |
| **Variables** | **Elasticity** | **CI** | **Absolute** | **%** | **Elasticity** | **CI** | **Absolute** | **%** | **Elasticity** | **CI** | **Absolute** | **%** | **Elasticity** | **CI** | **Absolute** | **%** |
| Age (years) |  |  |  |  |  |  |  |  |  |  |  |  |  |  |  |  |
| <20 | -0.58 | 0.178 | -0.044 | -53.73 | -0.305 | 0.263 | -0.066 | -55.703 | -0.031 | 0.021 | -0.001 | -0.332 | 0.011 | 0.097 | 0.003 | 1.429 |
| **Region** |  |  |  |  |  |  |  |  |  |  |  |  |  |  |  |  |
| Afar | -0.038 | -0.29 | 0.005 | 5.773 | -0.044 | -0.245 | 0.009 | 7.533 | -0.026 | -0.193 | 0.009 | 2.622 | -0.022 | -0.346 | 0.019 | 10.734 |
| Amhara | -2.169 | -0.091 | 0.084 | 103.028 | -1.292 | -0.036 | 0.039 | 32.733 | -1.011 | -0.11 | 0.2 | 57.312 | -0.458 | -0.009 | 0.01 | 5.81 |
| Oromia | -0.632 | 0.047 | -0.013 | -15.635 | -3.357 | -0.026 | 0.072 | 60.575 | -0.604 | 0.034 | -0.037 | -10.46 | -0.777 | 0.047 | -0.091 | -50.87 |
| Somali | -0.101 | -0.28 | 0.012 | 14.699 | -0.299 | -0.225 | 0.056 | 46.812 | -0.183 | -0.418 | 0.138 | 39.5 | -0.085 | -0.462 | 0.098 | 54.914 |
| B. Gumuz | -0.01 | -0.044 | 0 | 0.225 | -0.036 | 0.038 | -0.001 | -0.952 | -0.028 | -0.123 | 0.006 | 1.797 | -0.005 | -0.002 | 0 | 0.01 |
| SNNP | -0.706 | 0.058 | -0.017 | -21.34 | -0.986 | 0.091 | -0.074 | -62.316 | -0.114 | 0.073 | -0.015 | -4.282 | -0.307 | -0.094 | 0.072 | 40.233 |
| Gambela | 0.023 | 0.228 | 0.002 | 2.7 | -0.004 | 0.192 | -0.001 | -0.484 | -0.006 | 0.013 | 0 | -0.039 | -0.012 | 0.065 | -0.002 | -1.096 |
| Harar | -0.009 | -0.085 | 0 | 0.396 | -0.01 | -0.055 | 0 | 0.38 | 0.003 | 0.14 | 0.001 | 0.197 | -0.018 | -0.051 | 0.002 | 1.294 |
| Addis Ababa |  |  |  |  |  |  |  |  | 0.654 | 0.112 | 0.132 | 37.903 | 0.048 | 0.07 | 0.008 | 4.658 |
| Dire Dawa | 0 | -0.165 | 0 | 0.02 | 0.004 | -0.082 | 0 | -0.245 | -0.003 | 0.017 | 0 | -0.03 | -0.023 | -0.125 | 0.007 | 3.988 |
| **Partner education** |  |  |  |  |  |  |  |  |  |  |  |  |  |  |  |  |
| Primary | 0.454 | 0.137 | 0.027 | 32.461 | 0.31 | 0.105 | 0.027 | 22.542 | 0.696 | -0.113 | -0.143 | -40.791 | 0.53 | -0.261 | -0.345 | -192.77 |
| Secondary | 0.149 | 0.472 | 0.03 | 36.685 | 0.147 | 0.476 | 0.058 | 48.789 | 0.295 | 0.212 | 0.113 | 32.307 | 0.288 | 0.179 | 0.129 | 71.726 |
| Higher | 0.047 | 0.774 | 0.015 | 18.784 | 0.03 | 0.591 | 0.015 | 12.362 | 0.536 | 0.52 | 0.503 | 143.895 | 0.431 | 0.374 | 0.402 | 224.516 |
| **Religion** |  |  |  |  |  |  |  |  |  |  |  |  |  |  |  |  |
| Protestant | -0.016 | 0.092 | -0.001 | -0.758 | -0.456 | 0.112 | -0.042 | -35.6 | -0.407 | 0.081 | -0.059 | -16.909 | -0.053 | -0.031 | 0.004 | 2.278 |
| Muslim | 0.031 | -0.052 | -0.001 | -0.832 | 0.296 | -0.067 | -0.016 | -13.743 | -0.149 | -0.138 | 0.037 | 10.6 | -0.046 | -0.174 | 0.02 | 11.252 |
| Other | -0.005 | -0.06 | 0 | 0.148 | -0.099 | -0.161 | 0.013 | 11.029 | -0.058 | -0.072 | 0.008 | 2.158 | -0.014 | 0.285 | -0.01 | -5.76 |
| **Media exposure** |  |  |  |  |  |  |  |  |  |  |  |  |  |  |  |  |
| Yes | 0.49 | 0.213 | 0.045 | 54.475 | 0.11 | 0.255 | 0.023 | 19.446 | -0.212 | 0.118 | -0.045 | -12.882 | -0.222 | 0.111 | -0.061 | -34.299 |
| **Women education** |  |  |  |  |  |  |  |  |  |  |  |  |  |  |  |  |
| Primary | 1.099 | 0.562 | 0.263 | 322.01 | 0.55 | 0.491 | 0.223 | 187.852 | 0.726 | -0.129 | -0.169 | -48.493 | -0.015 | -0.341 | 0.013 | 6.996 |
| Secondary | 0.325 | 0.964 | 0.134 | 163.555 | 0.313 | 0.923 | 0.239 | 200.701 | 0.869 | 0.648 | 1.016 | 290.731 | 0.137 | 0.495 | 0.17 | 94.681 |
| **Women occupation** |  |  |  |  |  |  |  |  |  |  |  |  |  |  |  |  |
| Yes | 2.731 | 0.01 | 0.012 | 14.063 | 0.045 | 0.007 | 0 | 0.211 | 0.113 | -0.007 | -0.001 | -0.392 | -0.036 | -0.004 | 0 | 0.194 |
| **Partner occupation** |  |  |  |  |  |  |  |  |  |  |  |  |  |  |  |  |
| Yes | -0.062 | -0.001 | 0 | 0.035 | 1.227 | 0.004 | 0.004 | 3.237 | -6.021 | 0.006 | -0.061 | -17.509 | -0.445 | 0.006 | -0.007 | -3.856 |
| **Attitude toward violence** |  |  |  |  |  |  |  |  |  |  |  |  |  |  |  |  |
| Medium | 0.502 | 0.025 | 0.005 | 6.612 | -0.246 | 0.015 | -0.003 | -2.539 | 0.205 | -0.081 | -0.03 | -8.592 | 0.265 | 0.013 | 0.009 | 4.832 |
| High | 0.679 | 0.094 | 0.027 | 33.396 | -0.07 | 0.061 | -0.003 | -2.94 | 0.398 | 0.158 | 0.113 | 32.369 | 0.49 | 0.1 | 0.122 | 68.111 |
| **Social independence** |  |  |  |  |  |  |  |  |  |  |  |  |  |  |  |  |
| Medium | -0.105 | 0.139 | -0.006 | -7.633 | -0.079 | 0.158 | -0.01 | -8.731 | -0.261 | 0.061 | -0.029 | -8.175 | -0.216 | 0.002 | -0.001 | -0.703 |
| High | -0.167 | 0.323 | -0.023 | -28.227 | 0.03 | 0.346 | 0.009 | 7.264 | 0.028 | 0.467 | 0.024 | 6.82 | 0.163 | 0.383 | 0.156 | 87.083 |
| **Decision making** |  |  |  |  |  |  |  |  |  |  |  |  |  |  |  |  |
| Medium | 2.501 | 0.002 | 0.002 | 2.456 | 0.25 | -0.016 | -0.003 | -2.818 | -0.196 | -0.136 | 0.048 | 13.727 | 0.089 | -0.064 | -0.014 | -8.022 |
| High | 3.635 | 0.016 | 0.026 | 31.261 | 0.881 | 0.012 | 0.009 | 7.372 | 0 | 0.071 | 0 | -0.011 | 0.804 | 0.029 | 0.058 | 32.159 |
| **Birth order** |  |  |  |  |  |  |  |  |  |  |  |  |  |  |  |  |
| 2-3 | -0.716 | 0.052 | -0.016 | -19.336 | -0.378 | 0.059 | -0.018 | -15.474 | 0.141 | 0.07 | 0.018 | 5.147 | -0.028 | 0.057 | -0.004 | -2.208 |
| 4-5 | -0.299 | -0.045 | 0.006 | 7.011 | -0.42 | -0.089 | 0.031 | 25.918 | 0.113 | -0.159 | -0.032 | -9.228 | 0.275 | -0.189 | -0.13 | -72.584 |
| 6+ | -1.779 | -0.091 | 0.069 | 84.168 | -0.994 | -0.132 | 0.108 | 91.092 | -0.125 | -0.336 | 0.076 | 21.727 | 0.048 | -0.519 | -0.062 | -34.404 |
| **Wealth index** |  |  |  |  |  |  |  |  |  |  |  |  |  |  |  |  |
| Poorer | 0.731 | -0.063 | -0.02 | -24.121 | 0.625 | -0.069 | -0.036 | -29.905 |  |  |  |  |  |  |  |  |
| Middle | 1.38 | -0.024 | -0.014 | -17.02 | 0.753 | 0.016 | 0.01 | 8.615 |  |  |  |  |  |  |  |  |
| Richer | 1.937 | 0.139 | 0.115 | 140.927 | 1.275 | 0.133 | 0.14 | 118.088 |  |  |  |  |  |  |  |  |
| Richest | 0.776 | 0.359 | 0.119 | 145.353 | 0.351 | 0.23 | 0.067 | 56.116 | 0.962 | 0.042 | 0.074 | 21.037 | 1.749 | 0.04 | 0.176 | 98.251 |

***ANC4+ Antenatal care service four or more times**

**Table 4. Elasticities, concentration indices, and contributions of factors to disparity in SBA, 2011 and 2016 EDHS**

|  | **Rural** | | | | | | | | **Urban** | | | | | | | |
| --- | --- | --- | --- | --- | --- | --- | --- | --- | --- | --- | --- | --- | --- | --- | --- | --- |
|  | **2011** | | | | **2016** | | | | **2011** | | | | **2016** | | | |
| **Variables** | **Elasticity** | **CI** | **Absolute** | **%** | **Elasticity** | **CI** | **Absolute** | **%** | **Elasticity** | **CI** | **Absolute** | **%** | **Elasticity** | **CI** | **Absolute** | **%** |
| Age (years) |  |  |  |  |  |  |  |  |  |  |  |  |  |  |  |  |
| <20 | -3.499 | 0.15 | -0.084 | -211.936 | -0.055 | 0.27 | -0.011 | -6.828 | 0.039 | 0.128 | 0.01 | 2.357 | -0.061 | 0.126 | -0.024 | -7.558 |
| **Region** |  |  |  |  |  |  |  |  |  |  |  |  |  |  |  |  |
| Afar | -0.149 | -0.22 | 0.005 | 13.258 | -0.09 | -0.193 | 0.013 | 8.039 | -0.003 | -0.061 | 0 | 0.088 | -0.048 | -0.346 | 0.052 | 16.201 |
| Amhara | 2.389 | -0.105 | -0.04 | -101.069 | -1.576 | -0.078 | 0.094 | 56.509 | 0.315 | -0.207 | -0.132 | -30.408 | -0.322 | -0.048 | 0.049 | 15.06 |
| Oromia | 4.787 | 0.029 | 0.022 | 56.28 | -4.967 | -0.006 | 0.023 | 13.551 | 0.264 | 0.069 | 0.037 | 8.437 | -0.31 | 0.06 | -0.059 | -18.25 |
| Somali | 0.466 | -0.141 | -0.011 | -26.585 | -0.33 | -0.179 | 0.045 | 27.188 | 0.047 | -0.334 | -0.032 | -7.282 | -0.235 | -0.472 | 0.353 | 109.02 |
| B. Gumuz | 0.154 | -0.045 | -0.001 | -2.769 | -0.094 | 0.078 | -0.006 | -3.419 | 0.025 | -0.185 | -0.009 | -2.145 | -0.012 | 0.066 | -0.003 | -0.799 |
| SNNP | -1.154 | 0.067 | -0.012 | -31.215 | -1.557 | 0.089 | -0.106 | -63.66 | 0.133 | 0.209 | 0.056 | 12.977 | -0.428 | -0.05 | 0.067 | 20.863 |
| Gambela | 0.133 | 0.293 | 0.006 | 15.768 | -0.01 | 0.308 | -0.002 | -1.488 | 0.027 | 0.064 | 0.004 | 0.809 | -0.02 | 0.16 | -0.01 | -3.187 |
| Harar | 0.054 | -0.018 | 0 | -0.386 | -0.011 | 0.032 | 0 | -0.167 | 0.022 | 0.162 | 0.007 | 1.684 | -0.007 | -0.006 | 0 | 0.043 |
| Addis Ababa |  |  |  |  |  |  |  |  | 0.547 | 0.18 | 0.2 | 45.852 | 0.009 | 0.206 | 0.006 | 1.84 |
| Dire Dawa | 0.064 | -0.143 | -0.001 | -3.708 | -0.011 | -0.077 | 0.001 | 0.378 | 0.056 | -0.073 | -0.008 | -1.898 | -0.033 | -0.124 | 0.013 | 3.946 |
| **Partner education** |  |  |  |  |  |  |  |  |  |  |  |  |  |  |  |  |
| Primary | 1.843 | 0.135 | 0.04 | 100.635 | -0.192 | 0.104 | -0.015 | -9.232 | 0.53 | -0.072 | -0.078 | -17.844 | -0.02 | -0.244 | 0.015 | 4.761 |
| Secondary | 0.211 | 0.414 | 0.014 | 35.164 | 0.216 | 0.495 | 0.082 | 49.465 | 0.224 | 0.204 | 0.093 | 21.329 | 0.135 | 0.232 | 0.099 | 30.732 |
| Higher | -0.177 | 0.727 | -0.021 | -52.027 | 0.093 | 0.568 | 0.041 | 24.476 | 0.266 | 0.524 | 0.282 | 64.87 | 0.565 | 0.424 | 0.762 | 235.56 |
| **Religion** |  |  |  |  |  |  |  |  |  |  |  |  |  |  |  |  |
| Protestant | -1.6 | 0.092 | -0.024 | -59.554 | -0.51 | 0.118 | -0.046 | -27.754 | -0.268 | 0.162 | -0.088 | -20.303 | -0.112 | -0.01 | 0.003 | 1.075 |
| Muslim | -6.293 | -0.028 | 0.029 | 72.129 | 0.08 | -0.035 | -0.002 | -1.287 | -0.191 | -0.093 | 0.036 | 8.294 | 0.052 | -0.209 | -0.035 | -10.75 |
| Other | -0.571 | -0.061 | 0.006 | 14.053 | -0.165 | -0.139 | 0.018 | 10.553 | -0.027 | -0.056 | 0.003 | 0.703 | -0.016 | 0.301 | -0.015 | -4.721 |
| **Media exposure** |  |  |  |  |  |  |  |  |  |  |  |  |  |  |  |  |
| Yes | 0.19 | 0.136 | 0.004 | 10.456 | 0.111 | 0.208 | 0.018 | 10.718 | 0.551 | 0.165 | 0.185 | 42.47 | 0.467 | 0.166 | 0.247 | 76.264 |
| **Women education** |  |  |  |  |  |  |  |  |  |  |  |  |  |  |  |  |
| Primary | 1.711 | 0.729 | 0.199 | 503.48 | 0.615 | 0.68 | 0.322 | 193.162 | 0.496 | 0.168 | 0.169 | 38.804 | 0.084 | -0.143 | -0.038 | -11.824 |
| Secondary | 0.513 | 0.989 | 0.081 | 204.904 | 0.109 | 0.971 | 0.082 | 48.89 | 0.406 | 0.788 | 0.65 | 149.409 | 0.245 | 0.589 | 0.457 | 141.477 |
| **Women occupation** |  |  |  |  |  |  |  |  |  |  |  |  |  |  |  |  |
| Yes | 1.341 | 0.021 | 0.004 | 11.127 | 0.011 | 0.01 | 0 | 0.054 | -0.059 | 0.031 | -0.004 | -0.845 | -0.276 | 0.048 | -0.042 | -12.872 |
| **Partner occupation** |  |  |  |  |  |  |  |  |  |  |  |  |  |  |  |  |
| Yes | 38.696 | 0 | 0 | 0.955 | -0.975 | 0.007 | -0.005 | -3.147 | 0.722 | 0.007 | 0.01 | 2.393 | -1.268 | 0.015 | -0.062 | -19.144 |
| **Attitude toward violence** |  |  |  |  |  |  |  |  |  |  |  |  |  |  |  |  |
| Medium | -0.106 | 0.022 | 0 | -0.954 | -0.019 | 0.018 | 0 | -0.159 | 0.303 | -0.04 | -0.025 | -5.673 | 0.051 | 0.022 | 0.004 | 1.094 |
| High | 0.105 | 0.1 | 0.002 | 4.219 | 0.427 | 0.053 | 0.018 | 10.539 | 0.865 | 0.175 | 0.307 | 70.651 | -0.082 | 0.107 | -0.028 | -8.567 |
| **Social independence** |  |  |  |  |  |  |  |  |  |  |  |  |  |  |  |  |
| Medium | -1.437 | 0.123 | -0.028 | -71.263 | 0.016 | 0.15 | 0.002 | 1.094 | 0.418 | 0.072 | 0.061 | 13.946 | 0.119 | -0.02 | -0.008 | -2.377 |
| High | -0.504 | 0.321 | -0.026 | -65.239 | 0.112 | 0.251 | 0.022 | 12.965 | 0.32 | 0.456 | 0.296 | 68.122 | 0.455 | 0.388 | 0.561 | 173.389 |
| **Decision making** |  |  |  |  |  |  |  |  |  |  |  |  |  |  |  |  |
| Medium | 4.276 | 0.006 | 0.004 | 9.886 | 0.147 | -0.013 | -0.001 | -0.876 | 0.11 | -0.142 | -0.032 | -7.301 | -0.152 | -0.081 | 0.039 | 12.143 |
| High | 8.643 | 0.013 | 0.018 | 44.993 | 0.365 | 0.016 | 0.004 | 2.652 | 0.349 | 0.097 | 0.069 | 15.806 | -0.085 | 0.032 | -0.009 | -2.697 |
| **Birth order** |  |  |  |  |  |  |  |  |  |  |  |  |  |  |  |  |
| 2-3 | -9.251 | 0.019 | -0.029 | -72.137 | -0.947 | 0.049 | -0.036 | -21.432 | 0.106 | 0.026 | 0.006 | 1.274 | -0.497 | 0.039 | -0.062 | -19.079 |
| 4-5 | -9.169 | -0.064 | 0.094 | 237.794 | -1.252 | -0.093 | 0.09 | 53.809 | -0.011 | -0.185 | 0.004 | 0.992 | -0.256 | -0.302 | 0.246 | 75.928 |
| 6+ | -9.974 | -0.071 | 0.113 | 284.524 | -1.603 | -0.164 | 0.203 | 121.605 | -0.181 | -0.346 | 0.127 | 29.164 | -0.189 | -0.541 | 0.324 | 100.156 |
| **Wealth index** |  |  |  |  |  |  |  |  |  |  |  |  |  |  |  |  |
| Poorer | 3.805 | -0.046 | -0.028 | -71.118 | 1.231 | -0.024 | -0.022 | -13.47 |  |  |  |  |  |  |  |  |
| Middle | 4.023 | -0.012 | -0.008 | -19.414 | 1.235 | 0.025 | 0.024 | 14.262 |  |  |  |  |  |  |  |  |
| Richer | 6.09 | 0.152 | 0.148 | 372.987 | 1.245 | 0.122 | 0.117 | 70.116 |  |  |  |  |  |  |  |  |
| Richest | 2.857 | 0.304 | 0.139 | 351.053 | 0.439 | 0.24 | 0.081 | 48.615 | 1.806 | 0.064 | 0.236 | 54.144 | 0.564 | 0.069 | 0.124 | 38.42 |

***SBA Skilled Birth Attendance**

**Table 5. Elasticities, concentration indices, and contributions of factors to disparity in PNC, 2011 and 2016 EDHS**

|  | **Rural** | | | | | | | | **Urban** | | | | | | | |
| --- | --- | --- | --- | --- | --- | --- | --- | --- | --- | --- | --- | --- | --- | --- | --- | --- |
|  | **2011** | | | | **2016** | | | | **2011** | | | | **2016** | | | |
| **Variables** | **Elasticity** | **CI** | **Absolute** | **%** | **Elasticity** | **CI** | **Absolute** | **%** | **Elasticity** | **CI** | **Absolute** | **%** | **Elasticity** | **CI** | **Absolute** | **%** |
| **Age(years)** |  |  |  |  |  |  |  |  |  |  |  |  |  |  |  |  |
| <20 | -8.34 | 0.168 | -0.124 | -535.351 | 0.099 | 0.261 | 0.012 | 12.917 | 0.025 | 0.007 | 0 | 0.097 | 0.084 | 0.056 | 0.008 | 9.907 |
| **Region** |  |  |  |  |  |  |  |  |  |  |  |  |  |  |  |  |
| Afar | -0.441 | -0.29 | 0.011 | 48.936 | -0.075 | -0.245 | 0.009 | 9.105 | -0.009 | -0.193 | 0.002 | 0.966 | -0.035 | -0.346 | 0.022 | 25.69 |
| Amhara | -9.321 | -0.091 | 0.075 | 324.236 | -2.999 | -0.036 | 0.052 | 54.346 | -0.426 | -0.11 | 0.06 | 25.975 | -0.24 | -0.009 | 0.004 | 4.654 |
| Oromia | -8.328 | 0.047 | -0.035 | -150.926 | -7.579 | -0.026 | 0.093 | 97.831 | -0.477 | 0.034 | -0.021 | -8.887 | -0.567 | 0.047 | -0.048 | -56.668 |
| Somali | -0.402 | -0.28 | 0.01 | 43.045 | -0.312 | -0.225 | 0.033 | 34.979 | -0.079 | -0.418 | 0.042 | 18.27 | -0.061 | -0.462 | 0.051 | 59.852 |
| B.Gumuz | -0.423 | -0.044 | 0.002 | 7.173 | -0.143 | 0.038 | -0.003 | -2.712 | 0.031 | -0.123 | -0.005 | -2.138 | -0.03 | -0.002 | 0 | 0.101 |
| SNNP | -10.338 | 0.058 | -0.053 | -228.653 | -2.82 | 0.091 | -0.121 | -127.509 | 0.261 | 0.073 | 0.024 | 10.534 | -0.281 | -0.094 | 0.048 | 56.381 |
| Gambela | 0.191 | 0.228 | 0.004 | 16.6 | -0.026 | 0.192 | -0.002 | -2.524 | 0.007 | 0.013 | 0 | 0.052 | -0.022 | 0.065 | -0.003 | -3.072 |
| Harar | 0.071 | -0.085 | -0.001 | -2.308 | -0.01 | -0.055 | 0 | 0.26 | 0.016 | 0.14 | 0.003 | 1.258 | 0 | -0.051 | 0 | -0.014 |
| Addis Ababa |  |  |  |  |  |  |  |  | 0.287 | 0.112 | 0.041 | 17.886 | -0.122 | 0.07 | -0.016 | -18.269 |
| Dire Dawa | 0.036 | -0.165 | -0.001 | -2.252 | -0.016 | -0.082 | 0.001 | 0.666 | -0.001 | 0.017 | 0 | -0.005 | -0.037 | -0.125 | 0.008 | 9.806 |
| **Partner education** |  |  |  |  |  |  |  |  |  |  |  |  |  |  |  |  |
| Primary | -3.434 | 0.137 | -0.042 | -179.866 | 0.003 | 0.105 | 0 | 0.156 | 0.352 | -0.113 | -0.051 | -22.16 | 0.132 | -0.261 | -0.062 | -73.511 |
| Secondary | -0.438 | 0.472 | -0.018 | -79.01 | 0.093 | 0.476 | 0.021 | 22.082 | 0.678 | 0.212 | 0.184 | 79.965 | -0.188 | 0.179 | -0.061 | -71.721 |
| Higher | -1.255 | 0.774 | -0.086 | -370.987 | 0.232 | 0.591 | 0.065 | 68.368 | 0.793 | 0.52 | 0.528 | 228.923 | -0.247 | 0.374 | -0.167 | -197.047 |
| **Religion** |  |  |  |  |  |  |  |  |  |  |  |  |  |  |  |  |
| Protestant | 1.772 | 0.092 | 0.014 | 62.101 | -0.88 | 0.112 | -0.047 | -49.121 | -0.246 | 0.081 | -0.025 | -11.001 | 0.2 | -0.031 | -0.011 | -13.046 |
| Muslim | -8.593 | -0.052 | 0.039 | 169.562 | -0.128 | -0.067 | 0.004 | 4.257 | -0.169 | -0.138 | 0.03 | 12.944 | -0.007 | -0.174 | 0.002 | 2.734 |
| Others | 0 | -0.06 | 0 | 0 | -0.453 | -0.161 | 0.035 | 36.317 | -0.034 | -0.072 | 0.003 | 1.347 | 0.017 | 0.285 | 0.009 | 10.27 |
| **Media exposure** |  |  |  |  |  |  |  |  |  |  |  |  |  |  |  |  |
| Yes | -1.583 | 0.213 | -0.03 | -128.787 | 0.623 | 0.255 | 0.075 | 79.106 | 0.58 | 0.118 | 0.087 | 37.918 | 0.408 | 0.111 | 0.082 | 96.195 |
| **Women education** |  |  |  |  |  |  |  |  |  |  |  |  |  |  |  |  |
| Primary | 3.674 | 0.562 | 0.182 | 788.485 | 1.284 | 0.491 | 0.298 | 313.497 | 0.466 | -0.129 | -0.077 | -33.485 | 0.097 | -0.341 | -0.06 | -70.54 |
| Secondary | 4.932 | 0.964 | 0.419 | 1815.851 | 0.335 | 0.923 | 0.147 | 154.059 | 0.93 | 0.648 | 0.772 | 334.822 | 0.747 | 0.495 | 0.668 | 787.791 |
| **Women occupation** |  |  |  |  |  |  |  |  |  |  |  |  |  |  |  |  |
| Yes | 3.735 | 0.01 | 0.003 | 14.083 | -0.046 | 0.007 | 0 | -0.155 | -0.14 | -0.007 | 0.001 | 0.522 | 0.288 | -0.004 | -0.002 | -2.367 |
| **Partner occupation** |  |  |  |  |  |  |  |  |  |  |  |  |  |  |  |  |
| Yes | 140.575 | -0.001 | -0.014 | -58.795 | 3.371 | 0.004 | 0.006 | 6.364 | 0.101 | 0.006 | 0.001 | 0.315 | -0.889 | 0.006 | -0.01 | -11.769 |
| **Attitude toward violence** |  |  |  |  |  |  |  |  |  |  |  |  |  |  |  |  |
| Medium | -6.453 | 0.025 | -0.014 | -62.263 | -0.222 | 0.015 | -0.002 | -1.639 | 0.196 | -0.081 | -0.02 | -8.859 | 0.073 | 0.013 | 0.002 | 2.026 |
| High | 6.677 | 0.094 | 0.056 | 240.582 | -0.685 | 0.061 | -0.02 | -20.661 | 0.217 | 0.158 | 0.044 | 18.997 | -0.036 | 0.1 | -0.006 | -7.578 |
| **Social independence** |  |  |  |  |  |  |  |  |  |  |  |  |  |  |  |  |
| Medium | -8.602 | 0.139 | -0.106 | -457.472 | 0.563 | 0.158 | 0.042 | 44.401 | -0.15 | 0.061 | -0.012 | -5.063 | -0.032 | 0.002 | 0 | -0.16 |
| High | -1.199 | 0.323 | -0.034 | -147.969 | 0.272 | 0.346 | 0.045 | 46.796 | -0.033 | 0.467 | -0.02 | -8.545 | -0.056 | 0.383 | -0.038 | -45.309 |
| **Decision making** |  |  |  |  |  |  |  |  |  |  |  |  |  |  |  |  |
| Medium | 2.822 | 0.002 | 0 | 2.029 | 0.869 | -0.016 | -0.007 | -7.011 | -0.47 | -0.136 | 0.082 | 35.404 | 0.343 | -0.064 | -0.04 | -46.896 |
| High | -6.85 | 0.016 | -0.01 | -43.134 | 1.359 | 0.012 | 0.008 | 8.135 | -1.783 | 0.071 | -0.162 | -70.095 | 2.321 | 0.029 | 0.12 | 141.9 |
| **Birth order** |  |  |  |  |  |  |  |  |  |  |  |  |  |  |  |  |
| 2-3 | -20.019 | 0.052 | -0.091 | -395.601 | 0.13 | 0.059 | 0.004 | 3.807 | 0.218 | 0.07 | 0.02 | 8.543 | 0.676 | 0.057 | 0.069 | 81.64 |
| 4-5 | -23.387 | -0.045 | 0.093 | 401.843 | 0.679 | -0.089 | -0.029 | -29.965 | 0.075 | -0.159 | -0.015 | -6.616 | 0.348 | -0.189 | -0.119 | -140.259 |
| 6+ | -29.799 | -0.091 | 0.238 | 1032.096 | -0.198 | -0.132 | 0.012 | 12.954 | 0.114 | -0.336 | -0.049 | -21.233 | 0.206 | -0.519 | -0.193 | -227.55 |
| **Wealth index** |  |  |  |  |  |  |  |  |  |  |  |  |  |  |  |  |
| Poorer | -13.592 | -0.063 | 0.076 | 328.447 | 1.382 | -0.069 | -0.045 | -47.282 |  |  |  |  |  |  |  |  |
| Middle | -12.148 | -0.024 | 0.025 | 109.704 | 2.022 | 0.016 | 0.016 | 16.558 |  |  |  |  |  |  |  |  |
| Richer | -0.028 | 0.139 | 0 | -1.484 | 1.769 | 0.133 | 0.112 | 117.237 |  |  |  |  |  |  |  |  |
| Richest | 0.639 | 0.359 | 0.02 | 87.667 | 1.29 | 0.23 | 0.14 | 147.352 | 4.093 | 0.042 | 0.222 | 96.284 | 1.169 | 0.04 | 0.085 | 100.248 |

***PNC Postnatal care for the mother within 2 days of delivery**
