# Supplementary material for: Explaining changes in educational disparities in competent maternal health care services in urban and rural areas in Ethiopia
Source: Front Public Health. 2024 Apr 12;12:1332801. doi: 10.3389/fpubh.2024.1332801 (PMC11045905; doi:10.3389/fpubh.2024.1332801)
Supplement: Supplementary file 3 [file Image_1.pdf]

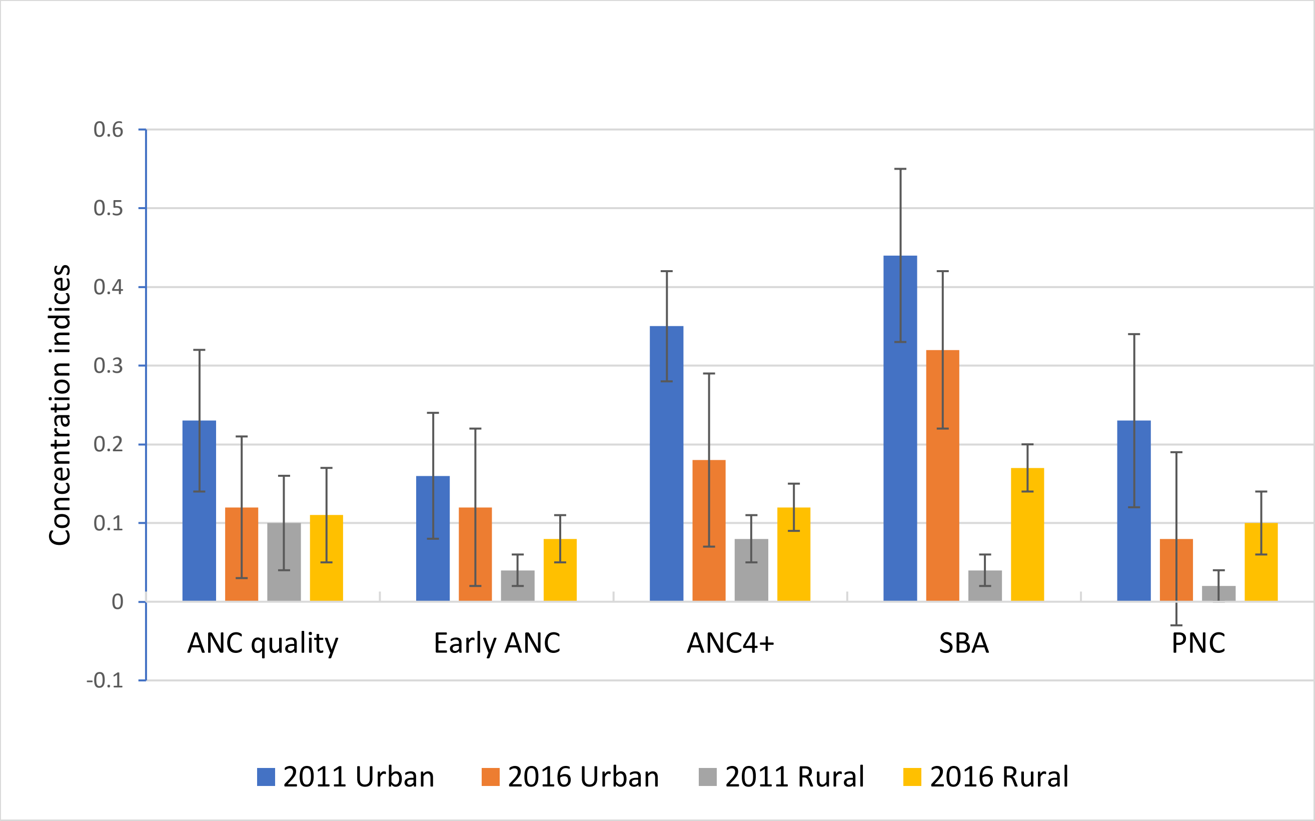

Fig. Erreygers concentration indices for the maternal health care services for urban and rural areas, 2011-2016 EDHS
